# Molecular mechanisms reconstruction from single-cell multi-omics data with HuMMuS

**DOI:** 10.1093/bioinformatics/btae143

**Published:** 2024-03-09

**Authors:** Remi Trimbour, Ina Maria Deutschmann, Laura Cantini

**Affiliations:** Institut Pasteur, Université Paris Cité, CNRS UMR 3738, Machine Learning for Integrative Genomics Group, F-75015 Paris, France; Institut de Biologie de l’Ecole Normale Supérieure, CNRS, INSERM, Ecole Normale Supérieure, Université PSL, 75005 Paris, France; Institut de Biologie de l’Ecole Normale Supérieure, CNRS, INSERM, Ecole Normale Supérieure, Université PSL, 75005 Paris, France; Institut Pasteur, Université Paris Cité, CNRS UMR 3738, Machine Learning for Integrative Genomics Group, F-75015 Paris, France; Institut de Biologie de l’Ecole Normale Supérieure, CNRS, INSERM, Ecole Normale Supérieure, Université PSL, 75005 Paris, France

## Abstract

**Motivation:**

The molecular identity of a cell results from a complex interplay between heterogeneous molecular layers. Recent advances in single-cell sequencing technologies have opened the possibility to measure such molecular layers of regulation.

**Results:**

Here, we present HuMMuS, a new method for inferring regulatory mechanisms from single-cell multi-omics data. Differently from the state-of-the-art, HuMMuS captures cooperation between biological macromolecules and can easily include additional layers of molecular regulation. We benchmarked HuMMuS with respect to the state-of-the-art on both paired and unpaired multi-omics datasets. Our results proved the improvements provided by HuMMuS in terms of transcription factor (TF) targets, TF binding motifs and regulatory regions prediction. Finally, once applied to snmC-seq, scATAC-seq and scRNA-seq data from mouse brain cortex, HuMMuS enabled to accurately cluster scRNA profiles and to identify potential driver TFs.

**Availability and implementation:**

HuMMuS is available at https://github.com/cantinilab/HuMMuS.

## 1 Introduction

Cells within a multicellular organism are remarkably heterogeneous, spanning many different molecular identities (Morris 2019). The molecular identity of a cell is the result of a complex interplay among different layers of molecular regulation, all of which can vary because of intrinsic and extrinsic factors. Recent advances in single-cell sequencing technologies have opened the possibility to measure such molecular layers of regulation, a.k.a. omics, at the resolution of the single cell. Examples of omics data currently accessible at single-cell resolution are chromatin accessibility (scATAC), methylation (snmC), expression (scRNA) (Method of the Year 2019: Single-cell multimodal omics 2020). In addition, sequencing technologies providing the joint profiling of multiple single-cell omics from the same cell have been developed (Mimitou *et al.* 2019, Lee *et al.* 2020). Examples of them are 10xGenomics Multiome platform, jointly profiling transcriptome and chromatin accessibility from the same cell, and CITE-seq, simultaneously quantifying cell surface proteins and transcriptome within a single cell (Stoeckius *et al.* 2017). All these data provide the unprecedented opportunity to reveal how different molecular layers interact through complex regulatory mechanisms to define cell identity.

Several methods, co-analysing single-cell omics data to elucidate the regulatory mechanisms that encode cellular identities, have been recently developed (Fleck *et al.* 2022, Jiang *et al.* 2022, Kartha *et al.* 2022, Skok Gibbs *et al.* 2022, Bravo González-Blas *et al.* 2023, Kamimoto *et al.* 2023, Ma *et al.* 2023). The output of these methods are Gene Regulatory Networks (GRNs), corresponding to graphs linking transcription factors (TFs) with their inferred target genes and/or peaks (Pratapa *et al.* 2020, Kang *et al.* 2021, McCalla *et al.* 2023). The GRNs are obtained by all methods performing TF–peak–gene associations based on binding motif databases [e.g. JASPAR (Castro-Mondragon *et al.* 2022)], then filtered through scRNA and scATAC data analysis. All these methods ignore intra-omics cooperation between biological macromolecules, which is crucial in biology. Indeed, TFs can cooperate in the regulation of gene expression by forming dimers and multiple DNA regions can co-regulate the expression of the same gene. In addition, state-of-the-art methods only consider TF–gene interactions present in binding motifs databases and miss all those interactions that are not reported there. Furthermore, all these methods infer GRNs by integrating scRNA and scATAC data, thus ignoring all other complementary layers of molecular regulation (e.g. methylation, proteome). Finally, many methods require either paired data, or perform cell pairing before GRN inference (Fleck *et al.* 2022, Jiang *et al.* 2022, Kartha *et al.* 2022, Ma *et al.* 2023). This is a major limitation, as paired single-cell multi-omics data are still rare and performing cell pairing in dataset profiled from different cells forces a decrease in the size of one of the two datasets thus reducing the richness of its information content.

Here, we introduce HeterogeneoUs Multilayers for MUlti-omics Single-cell data (HuMMuS), a flexible tool based on Heterogeneous Multilayer Networks (HMLNs) to reconstruct regulatory mechanisms from multiple single-cell omics data. HuMMuS considers not only inter-omics interactions (e.g. peak–gene, TF-peak), as done by the state-of-the-art, but also intra-omics ones (e.g. peak–peak, gene–gene, TF–TF) thus allowing to capture cooperation between biological macromolecules. This inclusion of intra-omics interactions allows HuMMuS to explore new TF–gene interactions not present in binding motif databases. In addition, HuMMuS is a flexible framework, that can be used both for paired and unpaired single-cell multi-omics data or easily extended to deal with additional omics data, thus not limiting the regulatory mechanisms analysis to only scRNA and scATAC, as it is currently done in the state-of-the-art.

We extensively benchmarked HuMMuS with respect to the state-of-the-art on four independent datasets of scRNA and scATAC. This benchmarking included the prediction of TF targets, TF binding regions, regulatory regions, and the association of its communities with known biological processes. Finally, by applying HuMMuS to unpaired scRNA, scATAC, and snmC data from mouse cortex, we showed that its GRN allows to accurately cluster scRNA profiles and to identify regulators relevant to mouse brain cortex.

HuMMuS is available at https://github.com/cantinilab/HuMMuS as R package, together with a tutorial for its usage.

## 2 Materials and methods

### 2.1 HuMMuS a new tool for molecular mechanisms reconstruction from single-cell multi-omics data

**Figure 1.**
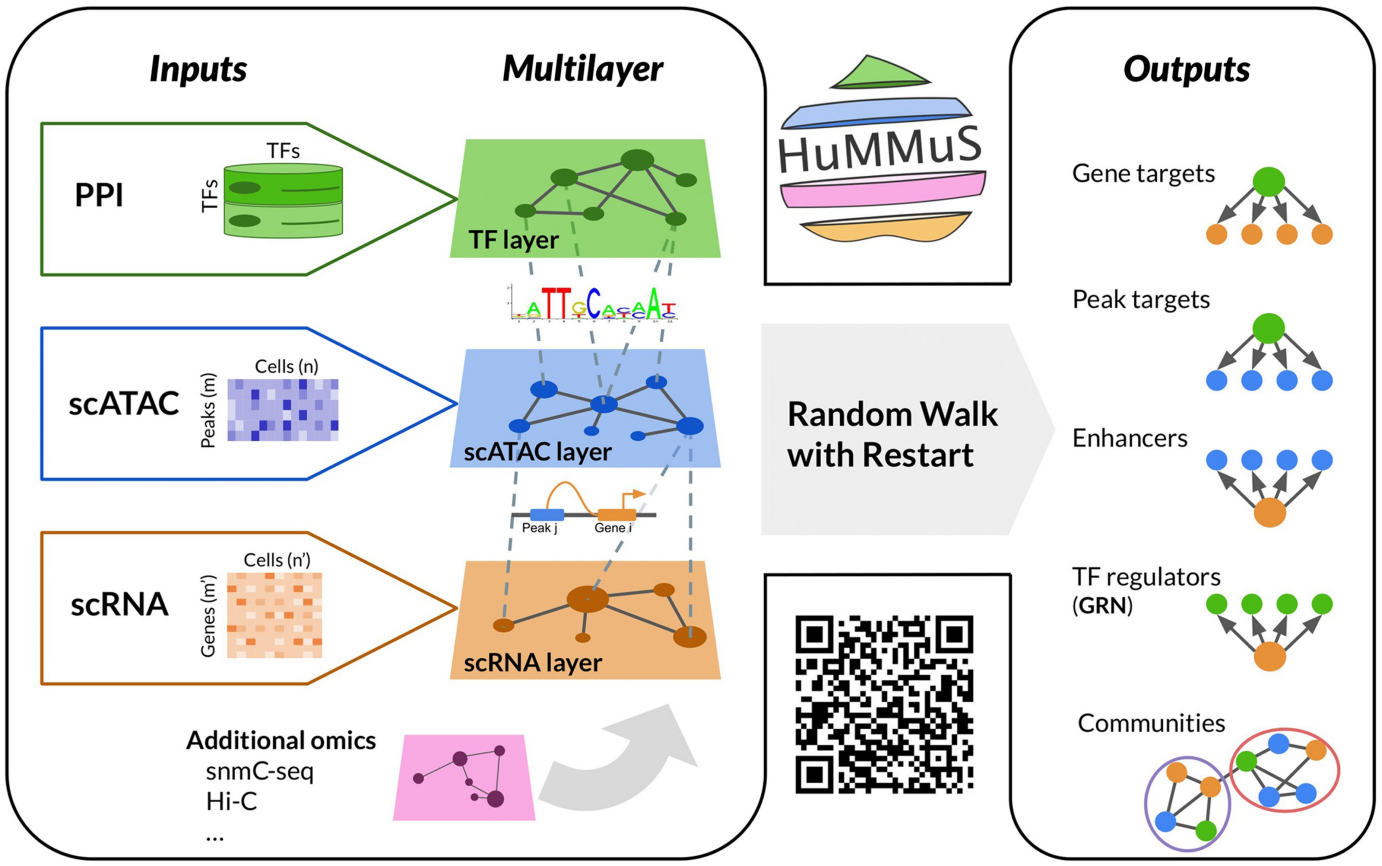
Schematic view of HuMMuS workflow.

We developed HeterogeneoUs Multilayers for MUlti-omics Single-cell data (HuMMuS), a new tool for regulatory mechanisms inference from single-cell multi-omics data (Fig. 1, https://github.com/cantinilab/HuMMuS).

HuMMuS is based on Heterogeneous Multilayer Networks (HMLNs). A HMLN is a network $M=(V_{m},E_{m},L),\;m=1,\ldots ,M$, composed of M, layers each of them containing different nodes $V_{m}\;$and different intra-layer links $E_{m}\subseteq V_{m}{\times V}_{m}$. Nodes of different layers are connected by inter-layers links encoded in **L** (Kivelä *et al.* 2014, Baptista *et al.* 2022). As summarized in Fig. 1, we reconstruct HMLNs composed of three layers: The TF layer, containing unlinked TFs, the scATAC layer containing peak co-accessibility information inferred from scATAC data and the scRNA layer encoding transcriptional regulation inferred from scRNA data. TF interactions were not considered here to compare HuMMuS fairly with respect to the state-of-the-art. An additional version of HuMMuS, called HuMMuS+TF, is also considered in the following to test the effect of TF–TF links on the performances. For all details on the layers’ construction see Supplementary Text. Of note, we here focused on this combination of omics data to not advantage HuMMuS by the additional information provided by other single-cell omics data. However, as the HMLN structure is flexible, HuMMuS can easily integrate other single-cell omics data, such as methylation (snmC) or Hi-C data, and additional information on known interactions, such as Protein-Protein interactions in the TF layer to capture TFs cooperativity. Once the HMLN is constructed, HuMMuS uses Random Walks with Restart (RWR) (Baptista *et al.* 2022) to mine the HMLN and extract different outputs: (i) the prediction of the targets of a TF, based on RWRs starting from each TF in the TF layer and exploring the full network until the scRNA layer; (ii) the prediction of the peaks bound by a given TF, based on RWRs starting from each TF in the TF layer and exploring the scATAC layer; (iii) the prediction of the regulatory regions (proximal and distal enhancers) associated to a given gene, based on RWRs starting in each gene of the scRNA layer and exploring the scATAC layer; (iv) the reconstruction of Gene Regulatory Networks (GRNs), based on RWRs starting in each gene of the scRNA layer and exploring the full network until the TF layer; (v) the extraction of communities in the GRN, reflecting tightly connected macromolecules in the HMLN frequently involved in the regulation of the same biological process or pathway (Barabási and Oltvai 2004). Of note, both the prediction of TF targets (output i) and the reconstruction of the GRNs (output iv), in principle lead to a TF–gene network. The choice of reconstructing GRNs by exploring the HMLN from genes to TFs is justified by the need of having a competition among different TFs in the regulation of a gene, as done in most of the GRN inference approaches (Pratapa *et al.* 2020, Kang *et al.* 2021, Jiang *et al.* 2022, Skok Gibbs *et al.* 2022, Fleck *et al.* 2022, Kartha *et al.* 2022, Bravo González-Blas *et al.* 2023, Kamimoto *et al.* 2023, Ma *et al.* 2023, McCalla *et al.* 2023). On the contrary, when predicting the targets of a TF, we want to treat each TF independently from the others and make genes compete among themselves.

For this reason, we obtain the output (i) by exploring the HMLN from TFs to genes. See Supplementary Table S1 and Supplementary Fig. S1 for a computational comparison between the two approaches and methods for all details on the parameter choice for the RWR and the possible outputs.

Thanks to the use of a HMLN structure, HuMMuS has multiple advantages with respect to the state-of-the-art. First, it captures not only inter-omics interaction (e.g. peak–gene, TF-peak), as done by the state-of-the-art, but also intra-omics ones (e.g. peak–peak, gene–gene, TF–TF). This allows HuMMuS to capture cooperation between biological macromolecules and use it to predict, e.g. TF–gene interactions not present in binding motifs databases. In addition, HuMMuS is a flexible framework, that can be used both for paired and unpaired single-cell multi-omics data or easily extended to deal with additional omics data, thus not limiting the regulatory mechanisms analysis to only scRNA and scATAC, as it is currently done in the state-of-the-art.

In the following we extensively benchmark HuMMuS against SCENIC+, CellOracle and Pando (Fleck *et al.* 2022, Bravo González-Blas *et al.* 2023, Kamimoto *et al.* 2023), being the most famous published works in the field. Interestingly, CellOracle is the only existing method considering some cooperation at the peaks level. In addition, we included GENIE3 (Huynh-Thu *et al.* 2010) in the benchmark as a baseline for performances when considering scRNA alone. All the benchmarking is performed on four test cases (see Supplementary Text and Supplementary Table S2): two datasets (called in the following Chen and Liu) of human Embryonic Stem Cells (hESCs), jointly profiled for scRNA and scATAC (i.e. paired data), and two unpaired scRNA and scATAC datasets of mouse Embryonic Stem Cells (mESCs) (called in the following Duren and Semrau). For details on HuMMuS layers structure in these four datasets see Supplementary Table S3. Of note, in Duren and Semrau, being the data unpaired, the scRNA and scATAC information has been profiled from different cells all extracted from mESCs. These last two test cases thus allow to test the impact of cell pairing on the performances of the different methods. The choice of these four test cases is justified by the availability of ChIP-seq and TF perturbation experiments in hESCs and mESCs from McCalla *et al.* (2023). These additional data, already used in benchmarking works (McCalla *et al.* 2023), allow indeed to build good ground truths for the different tests presented in the following sections.

## 3 Results

### 3.1 HuMMuS outperforms the state-of-the-art in TF target prediction

We first focused on benchmarking HuMMuS with respect to the state-of-the-art based on the quality of its TF targets predictions. This analysis has been performed on the four test cases presented above, corresponding to scRNA and scATAC profiling of hESCs and mESCs. As ground truth of the TF-targets interactions we used the intersection between ChIP-seq and TF perturbations experiments, as done in (McCalla *et al.* 2023). This choice represents indeed the best estimation of TF targets we can get for real data, as it assures the presence of a binding site for the TF on the promoter of the target gene and, at the same time, a downregulation of the target gene once the TF is knocked down/out.

**Figure 2.**
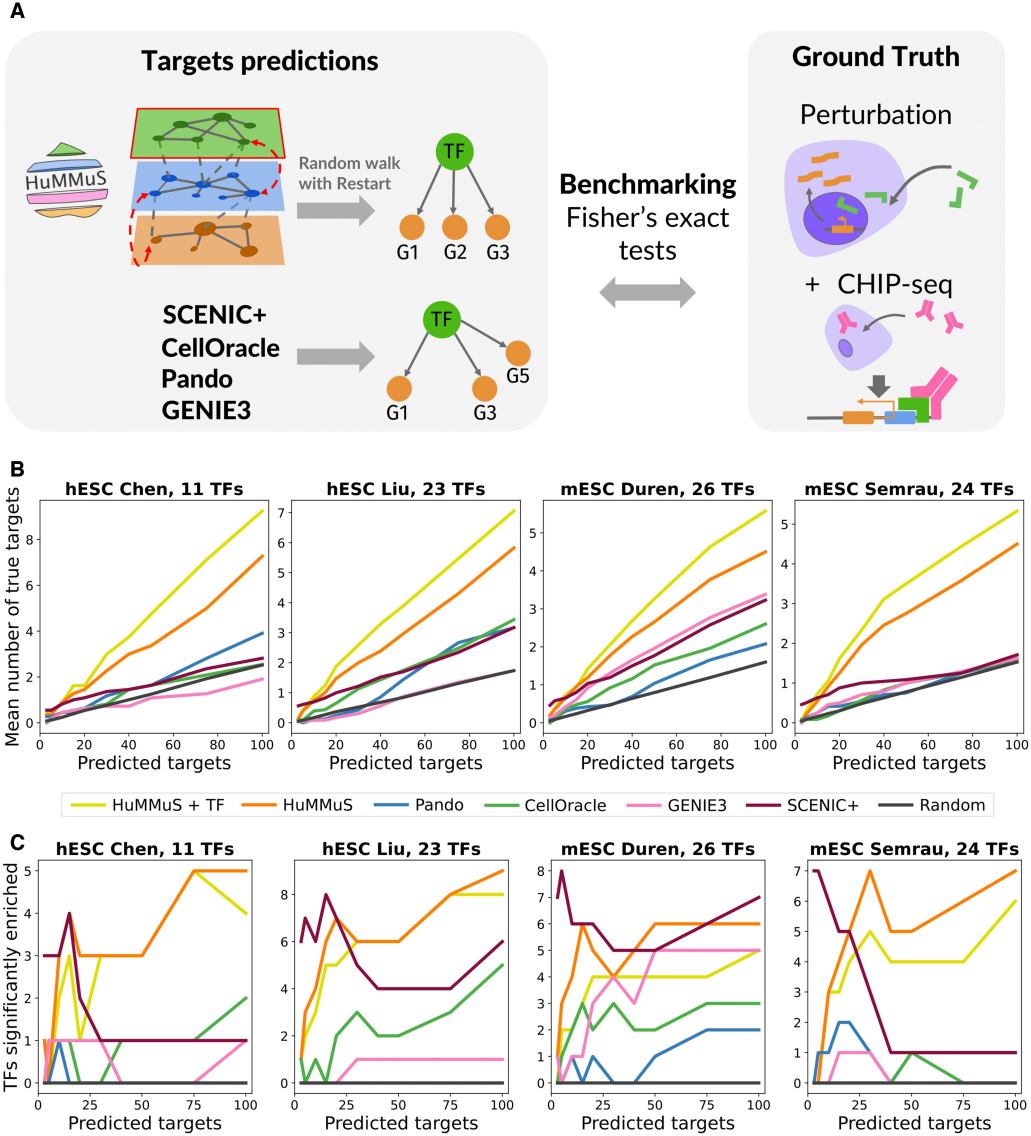
Transcription Factor (TF) targets prediction benchmarking. (A) schematic view of the performed benchmarking. (B) average number of correctly predicted targets per TF. (C) number of TFs having a significant amount of correctly predicted targets (Fisher’s exact test *P*-value <0.05). In (B and C) results for different methods are provided: *HuMMuS +TF*, HuMMuS, SCENIC+, Pando, CellOracle, GENIE3, and random.

As described in Fig. 2A, in each of the four test cases, HuMMuS and the other state-of-art algorithms have been independently applied, a ranking of putative targets for each TF is then identified and compared with the ground truth described above. The ranking of putative gene targets for a TF is obtained for the state-of-the art methods as the list of genes linked to the TF. The genes are ordered according to the weight of their links. For HuMMuS instead, we perform a Random Walk with Restart (RWR) starting from each TF and going across all the HMLN, thus obtaining a ranking of putative target genes based on their closeness to the TF. The overlap for all methods with the ground truth is then analyzed when cutting the ranking at different levels (3, 5, 10, 15, 20, 30, 40, 50, 75, 100).

As shown in Fig. 2B, HuMMuS outperforms the state-of-the-art in all the four tested datasets at every threshold, except when focusing on the very top of the ranking (3–5 first inferred TF–gene links), where SCENIC+ shows better performances. In addition, the performances of HuMMuS get further improved once including TF–TF interactions in the network (*HuMMuS+TF*). In Semrau the results of state-of-the-art methods are close to random, here represented with a black curve. Of note, even when pairing the cells in the two unpaired datasets, the performances observed for HuMMuS are not affected (see Supplementary Fig. S2). To then test whether the observed performances were driven by a subgroup of TFs or consistent for a high number of them, we computed the number of TFs having a significant number of targets in their top predicted targets (see Supplementary Text for details). As shown in Fig. 2C, overall, all methods get few TFs with a significant amount of correctly predicted targets. In this test too, HuMMuS gets the best performances in three out of four test cases. Taken together these two results suggest a high potential for HuMMuS in TF targets prediction.

### 3.2 HuMMuS outperforms the state-of-the-art in regulatory region identification

We then benchmarked HuMMuS with respect to the state-of-the-art based on known regulatory regions identification. This benchmark was realized in two steps: first, the ability to predict the peaks bound by a TF is tested; then, the quality of the regulatory regions (proximal and distal enhancers) predicted for each gene is evaluated. As GENIE3 does not provide any information on regulatory regions, it was excluded from this part of the benchmarking.

**Figure 3.**
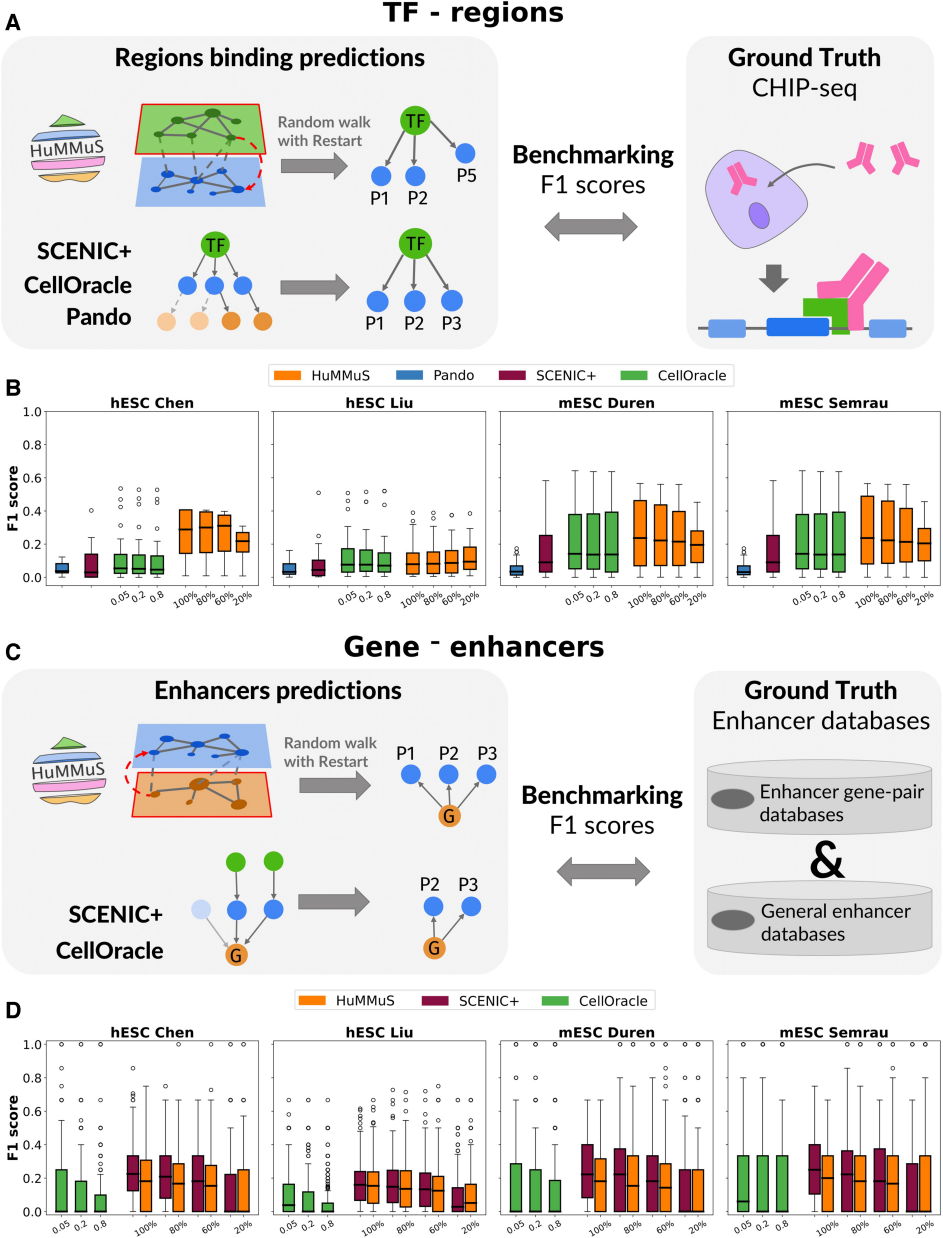
Regulatory regions benchmarking. (A) schematic view of the benchmarking performed for TF-peak associations. (B) F1 score of the intersection between the ground-truth TF-peak associations and those inferred by Pando, CellOracle, SCENIC+ and HuMMuS; the 100%, 80%, 60%, 20% thresholds of HuMMuS correspond to the number of nodes retained from the predictions. For CellOracle instead, 0.05, 0.2, and 0.8 correspond to the correlation thresholds of the model, with 0.8 being the default one. (C) schematic view of the benchmarking performed for gene-peak associations. (D) F1 score of the intersection between the ground-truth gene-peak associations and those inferred by CellOracle, SCENIC+ and HuMMuS. In (B, D) results for different methods are provided: HuMMuS, SCENIC+, Pando, CellOracle. The thresholds are the same as those of panel (B).

As shown in Fig. 3A, to test the quality of the peaks associated with a TF, in HuMMuS we used RWRs from each TF as a proxy of the compatibility between a TF and peaks and filtered the obtained peak ranking at different levels (100%, 80%, 60%, 20%). For SCENIC+, CellOracle and Pando instead, we considered the peaks retained by the model as associated with each TF (see Supplementary Text for details). In CellOracle different peak co-accessibility correlation thresholds have been considered 0.05, 0.2, and 0.8, with the last being the default threshold. We finally compared the predictions obtained by the various methods with the ground-truth composed of ChIP-seq experiments results on the biological system under analysis (mESCs and hESCs) from Hammal *et al.* (2022).

See Supplementary Text for further details on the analysis.

Overall, as shown in Supplementary Fig. S3A, HuMMuS identifies more peaks associated with a TF than alternative methods. This result is not surprising as, differently from the state-of-the-art, HuMMuS leverages all the peak layer without constraints neither on genomic windows nor on known TF motifs. This choice of considering TF-peak interactions outside of TF binding motif databases allows to include interactions that are missing in such databases and situation where, due to cooperation between TFs (e.g. condensates), there is a modification in the binding region (Guo and Gifford 2017, Kribelbauer *et al.* 2023). More interestingly, as shown in Fig. 3B, once checking the quality of the identified TF-peak associations based on F1 score, HuMMuS outperforms the state-of-the-art in three out of four datasets and it performs comparably to CellOracle in the fourth dataset. Regarding the percentage of true positives (Supplementary Fig. S3B), HuMMuS and CellOracle are among the best performant methods in three out of four datasets, once focusing on comparable numbers of tested predictions (1%–10% filtering of HuMMuS). On the other hand, SCENIC+ is among the best performing methods in only one dataset out of four. Overall, these results suggest that considering peak co-accessibility favorably helps reconstruction of TF-peak interactions.

We then focused on the regulatory regions associated with each gene. As shown in Fig. 3C, in HuMMuS, peaks are ranked based on the RWR starting from the gene. For CellOracle and SCENIC+ instead, the model directly provides a set of peaks associated to a gene. Regarding thresholding, HuMMuS and SCENIC+ were filtered to have a comparable number of predictions (see Supplementary Text), while CellOracle was filtered with different correlation thresholds: 0.05, 0.2, and 0.8, with the last being the default one. The obtained predictions were finally compared with a ground truth composed of gene-regulatory regions associations available from different databases (Visel *et al.* 2007, Forrest *et al.* 2014, Naville *et al.* 2015, Bai *et al.* 2020, Clément *et al.* 2020, Gao and Qian 2020, Moore *et al.* 2020). For all details on the analysis, see Supplementary Text. GENIE3 and Pando have been excluded from this analysis as they did not provide an output allowing for this type of evaluation.

As shown in Supplementary Fig. S4A, overall HuMMuS gets more enhancers associated with each gene. Again, this result is not surprising given that the intrinsic structure of HuMMuS allows it to predict new peak–gene associations, without genomic windows constraints. In addition, as shown in Fig. 3D HuMMuS and SCENIC+ comparably overperform CellOracle. Same results apply when considering the percentage of true positive (Supplementary Fig. S4B). Overall, the obtained results indicate that the enhancers predicted by HuMMuS and SCENIC+ tend to more frequently reflect known ones.

Taken together these two results suggest that HuMMuS can powerfully predict regulatory regions associated with TFs or genes. Also in this case, the results observed for HuMMuS in the two unpaired data (Duren and Semrau) are not affected by cell pairing (Supplementary Fig. S5).

### 3.3 HuMMuS outperforms the state-of-the-art in the biological relevance of its gene communities

We benchmarked HuMMuS with respect to the state-of-the-art based on the biological relevance of their gene communities. Indeed, gene communities in biological graphs have been previously shown to frequently reflect known pathways and biological processes (Barabási and Oltvai 2004, Cantini *et al.* 2015, Choobdar *et al.* 2019).

**Figure 4.**
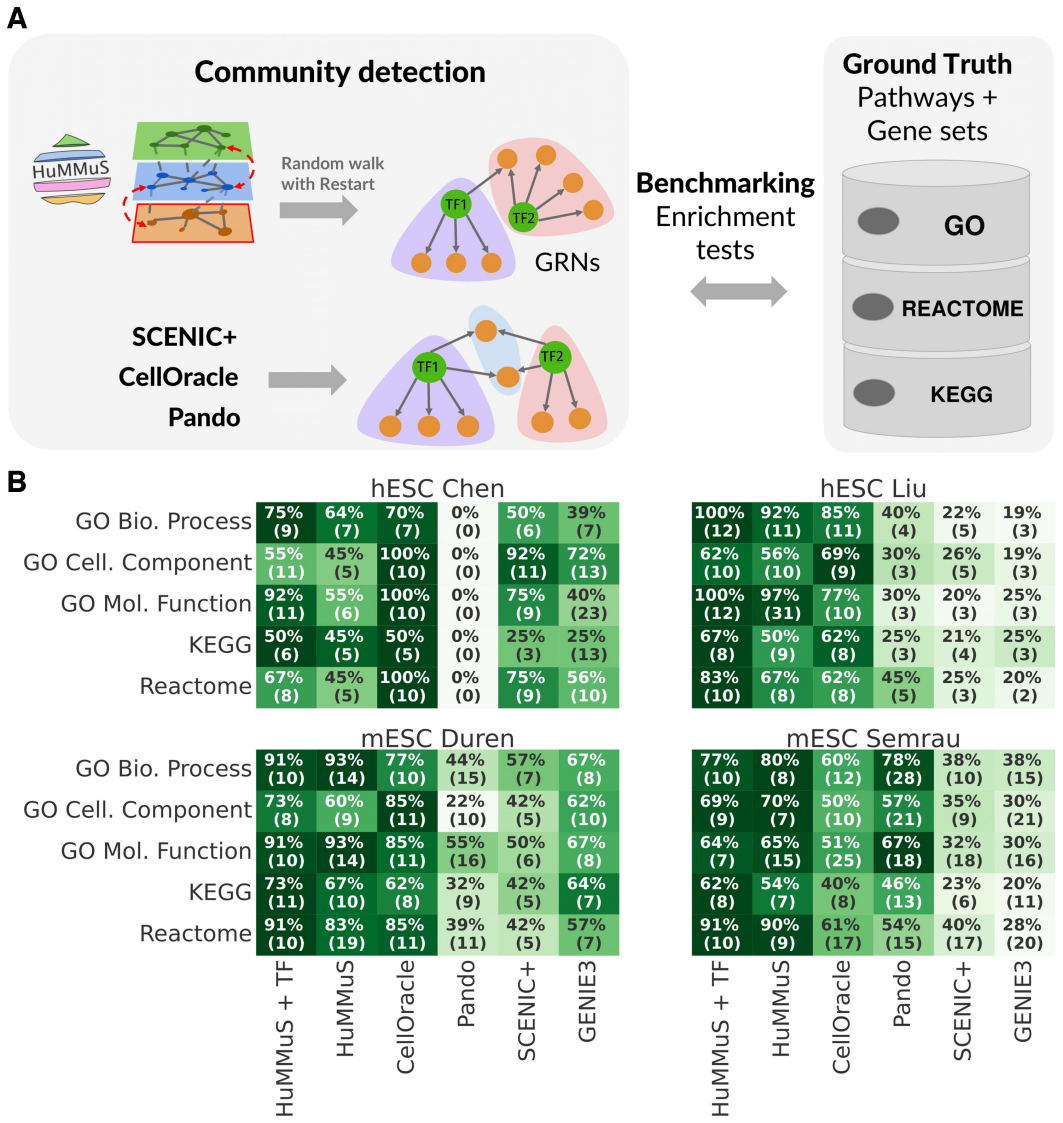
Community detection benchmarking. (A) Schematic view of the benchmarking performed for community detection. (B) Heatmaps of percentage of enriched communities in each benchmarked method across the five biological databases. The values reported in the table correspond to the percentage of enriched communities, while those in parentheses are the actual number of enriched communities.

As shown in Fig. 4A, the Louvain algorithm (Blondel *et al.* 2008) was applied to the HuMMuS GRN and to those of the state-of-the-art and the biological relevance of the obtained communities was evaluated based on the percentage of communities enriched in pathways [KEGG (Kanehisa and Goto 2000, Kanehisa *et al.* 2023) and REACTOME (Gillespie *et al.* 2022)] and Gene Ontologies (Ashburner *et al.* 2000, Gene Ontology Consortium 2021). Before running community detection, as most of the GRNs are highly dense (density > 0.8 in half of networks see Supplementary Table S4), a filtering was applied to the links to make all networks equally dense. Regarding the community detection, as the Louvain algorithm depends on the resolution parameter, we here run it with resolution varying in the range 0–2 and choose for each method the resolution giving best performances and a reasonable number of communities (≥10). See Supplementary Text for details on the analysis, Supplementary Table S5 for performances across different resolution values.

Figure 4B shows the results of the comparison. Regarding the number of enriched communities, all methods vary in a range of 5–31 communities, depending on the test case and the database under analysis. Concerning the enrichment in pathways and Gene Ontologies, in three out of four test cases (Liu, Duren, and Semrau), HuMMuS gets the highest percentage of enriched communities in most of the databases. Interestingly, in two out of these three datasets HuMMuS performances get even better once including TF–TF links (see HuMMuS + TF in Fig. 4B). In the remaining test case (Chen), CellOracle gets better results. Of note, no evident correlation emerges between the number of identified communities and the performances of the different methods (see Supplementary Table S5).

### 3.4 HuMMuS is robust to unbalanced cell type proportions across omics

Most of the state-of-the-art methods for GRN inference in single-cell multi-omics data require paired data. This requirement is due, on one side, to the use of regression models to infer the interactions, which intrinsically requires paired data, and, on the other side, to the fact that different cell type proportions might impact GRN inference. As HuMMuS is here proposed as a tool that can deal with unpaired data, we evaluated its robustness with respect to unbalanced cell type proportions across omics. For this we employed scRNA (Saunders *et al.* 2018) and scATAC (atac_v1_adult_brain_fresh_5k—Datasets—Single Cell ATAC—Official 10x Genomics Support) data profiled from mouse cortical neurons. We only considered three cell populations: MGE, Layer 2/3 and Layer 6; corresponding to a total of 1143 cells. We then tested four scenarios (i) full datasets; (ii) half scRNA cells for Layer 2/3 and everything else unaltered; (iii) half scATAC cells for Layer 6 and everything else unaltered, and (iv) half scRNA cells for Layer 2/3, half scATAC cells for Layer 6 and everything else unaltered. We then used HuMMuS to construct GRNs for all the four scenarios and computed the Spearman correlation between the full dataset (scenario 1) and all others. As shown in Supplementary Fig. S6, such correlations resulted to be 0.91–0.95, indicating a robustness of HuMMuS to different cell type propositions across different omics, thus making it particularly suitable for unpaired single-cell data.

Of note, as shown in Supplementary Fig. S6, we do not observe the same robustness in the individual layers (Spearman correlations of 0.66–0.68). Thus, further suggesting that the use of RWRs helps to compensate for false and/or missing links in the single layers.

### 3.5 Challenging HuMMuS in mouse cortex profiled for scRNA, scATAC, and snmC

We finally challenged HuMMuS in the reconstruction of molecular mechanisms of the mouse brain cortex. Differently from the state-of-the-art, here for the first time we take into account three single-cell omics data: scRNA (Saunders *et al.* 2018), scATAC (atac_v1_adult_brain_fresh_5k—Datasets—Single Cell ATAC—Official 10x Genomics Support), and snmC (Luo *et al.* 2017). The data of size 55 803 cells in scRNA, 2317 cells in scATAC and 3386 cells in snmC are unpaired, obtained by profiling mouse cortical neurons.

**Figure 5.**
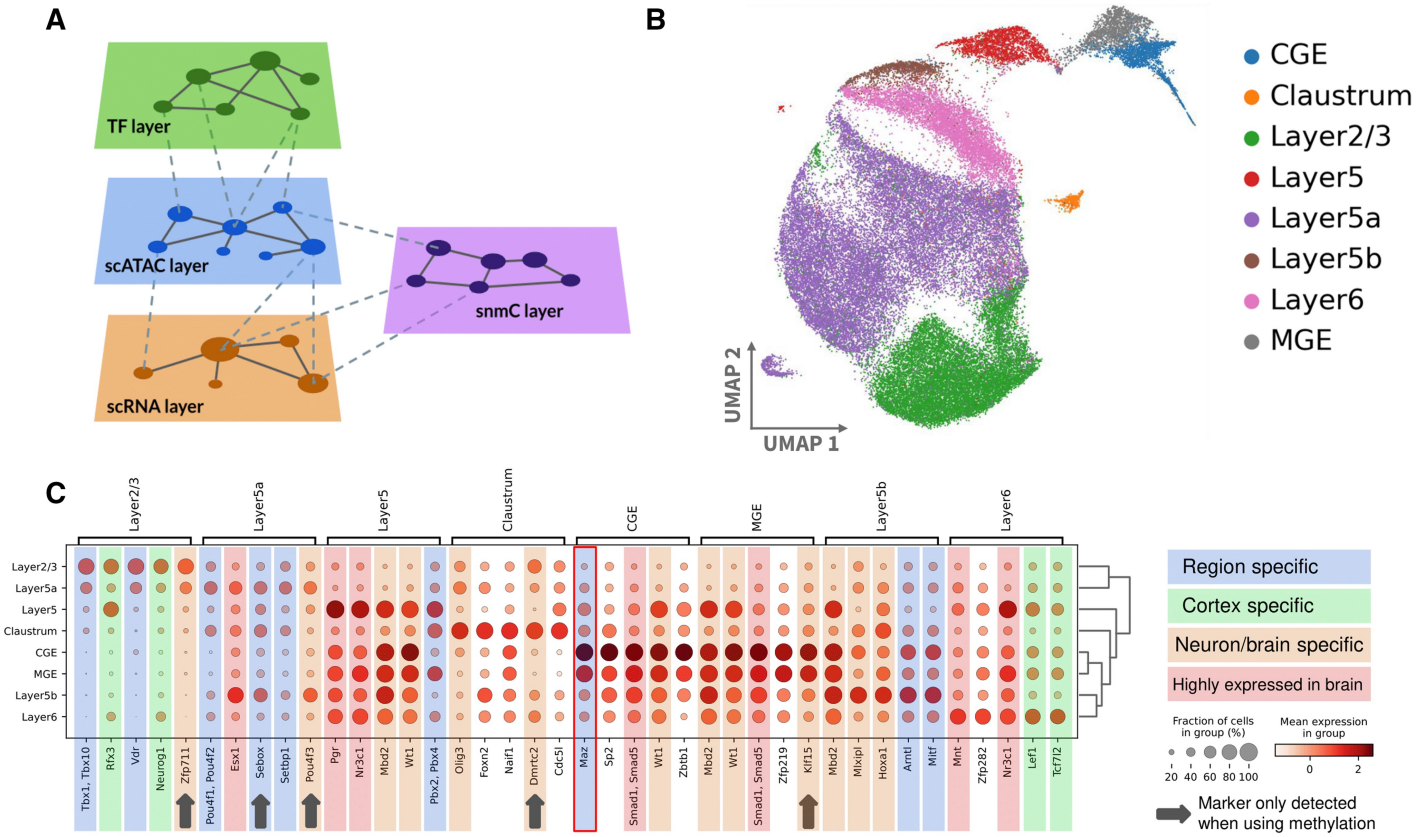
Challenging HuMMuS on scRNA, scATAC and snmC from mouse cortex. (A) HMLN used in HuMMuS to reconstruct regulatory mechanisms from scRNA, scATAC and snmC. (B) UMAP plot obtained from HuMMuS regulon activity. Cells are colored according to the labels present in their original publication and in previous analyses (Saunders *et al.* 2018, Cao and Gao 2022). (C) Heatmap of activity for the top five TFs per cell population. Colors are used to denote the type of validation available; arrows indicate TFs lost once methylation is excluded from the analysis.

Following the HuMMuS pipeline, we reconstructed two HMLNs, one composed of four layers (TF layer, scATAC layer, snmC layer, and scRNA layer; see Fig. 5A) and one composed of three layers (TF layer, scATAC layer, and scRNA layer). The second HMLN is intended to test the added value brought by methylation in the analysis. Then RWRs from the scRNA layer have been used to extract a GRN composed of 637 regulons, each corresponding to a TF and its associated genes ranked by the strength of association (Badia-i-Mompel *et al.* 2022).

As a first observation, the activity of the obtained regulons, computed according to Badia-i-Mompel *et al.* (2022) and Teschendorff and Wang (2020), is able to correctly cluster the cells according to their area of origin in the mouse cortex (see Fig. 5B). This suggests that the regulons identified by HuMMuS can nicely recapitulate the known heterogeneity present between the analyzed cells and already reported in Saunders *et al.* (2018) and Cao and Gao (2022). These conclusions apply with and without the additional methylation layer (See Supplementary Fig. S7A).

We then focused on the results obtained with HuMMuS when methylation is included in the multilayer. We then validate in the literature the top five differentially active regulons associated to each cell population (Fig. 5C, Supplementary Text for details). Of the obtained 34 regulons, 76% of their TFs have an already reported association with either neurons, cortex, or brain (see Supplementary Table S6). In particular, five of them (Esx1, Pgr, Nr3C1, Smad1/5, Mnt) are reported in the Bgee database as expressed in the brain (Bastian *et al.* 2021). Nine of them Zfp711 (Kleine-Kohlbrecher *et al.* 2010), Pou4f3 (Zou *et al.* 2012), Mbd2 (Hendrich and Bird 1998), Wt1 (Dame *et al.* 2006), Olig3 (Müller *et al.* 2005), Dmrtc (Casado-Navarro and Serrano-Saiz 2022), Mlxipl (Russ *et al.* 2021), Hoxa1 (Gavalas *et al.* 1997) are documented in publications associating them with either brain or neurons and thirteen of them [Tbx1/Tbx10 (Flore *et al.* 2017), Rfx3 (Callaway *et al.* 2021, Bravo González-Blas *et al.* 2023), Neurog1 (Dixit *et al.* 2014), Vdr (Gezen-Ak *et al.* 2011), Pou4f1/Pou4f2 (Turner *et al.* 1994), Sebox (Cinquanta *et al.* 2000), Setbp1(Cardo *et al.* 2023), Pbx2/Pbx4 (Golonzhka *et al.* 2015), Maz (Wang *et al.* 2013, Ning *et al.* 2015), Arntl (Okano *et al.* 2001), Mitf (Ohba *et al.* 2016), Lef1 (Nagalski *et al.* 2013), Tcf7l2 (Nagalski *et al.* 2013)] are reported in publications specifically referring to the mouse cortex. Of note, four of these TFs were also already documented to be associated to the specific region of the cortex where HuMMuS found them to be differentially active. This is the case for Rfx3 and Neurog1, that we find associated with Layer 2/3 and that had been previously associated with this exact brain region (Dixit *et al.* 2014, Gray *et al.* 2017, Callaway *et al.* 2021, Bravo González-Blas *et al.* 2023). In addition, Lef1 and Tcf17l2 have been documented to be associated with deep layers of the cortex and HuMMuS identifies them in layer 6 (Nagalski *et al.* 2013).

Finally, HuMMuS suggests the possible regulatory role of MAZ in CGE-derived cortical inhibitory interneurons. Through bibliographic research MAZ is documented to have a role in neuronal stem cells differentiation and as potential regulator in Purkinje cells, a GABAergic inhibitory neuron population (Wang *et al.* 2013, Ning *et al.* 2015). HuMMuS associates it to the Caudal Ganglionic Eminence (CGE) region, producing a high proportion of cortical inhibitory neurons (30%) (Williams and Riedemann 2021). In addition, in the top 10% of the 9341 inferred targets of MAZ, we can find Cntnap3, Dlx5, Sp9, Dlx6, Nr2c2ap, Dlx2, Arx, Grik3, all genes documented to be differentially expressed in inhibitory interneurons in The Mouse Organogenesis Atlas (MOCA) (Cao *et al.* 2019).

Once methylation is excluded, five TFs are lost: Zfp711 in Layer 2/3, Sebox and Pou4f3 in Layer 5a, Dmrtc2 in Claustrum and Klf15 in MGE. Of note, Zfp711, Pou4f3 and Dmrtc2 had been validated on existing literature to be neuron/brain specific, while Sebox had been validated in the literature to be associated with Layer 5a neurons. The five regulons that are lost once excluding methylation are replaced by the following TFs: Trp63 for Layer 2/3, Myt1l and Olig3 for Layer 5a, Hoxb2 in Claustrum and Plag1 in MGE. Of them, Olig3 (Müller *et al.* 2005), Plag1 (Alam *et al.* 2005), and Myt1l (Mall *et al.* 2017) have been previously associated with neurons/brain and Hoxb2 (Davenne *et al.* 1999) is a known marker of Claustrum. Altogether these results suggest that methylation has an impact on the selection of the differentially active regulons associated to each cell population. However, whether such effect is an improvement or not, depends on the cell population under analysis. Indeed, the selection of TFs in Layer 2/3 and Layer 5a improves when methylation is considered, while for Claustrum and MGE the quality of the regulons is higher when methylation is excluded.

## 4 Discussion

Cell identities result from the joint activity of different molecular layers of regulation. These molecular layers can be measured nowadays thanks to single-cell sequencing technologies, such as scRNA, scATAC, and snmC.

Different methods have been recently designed to reconstruct molecular mechanisms from different single-cell omics data. Here we proposed HeterogeneoUs Multilayers for MUlti-omics Single-cell data (HuMMuS), a flexible tool based on Heterogeneous Multilayer Networks (HMLNs) to reconstruct regulatory mechanisms from multiple single-cell omics data. HuMMuS is found to have better performance than the state-of-the-art in the prediction of TF targets, TF binding regions, regulatory regions and in the identification of biologically relevant gene communities. Once applied to the integration of scRNA, scATAC, and snmC data profiled from mouse cortex, HuMMuS identified relevant regulatory mechanisms.

Overall, the main advantages of HuMMuS are the ability to capture intra-omics cooperation between biological macromolecules and its flexibility, allowing to easily integrate additional omics or prior information (e.g. pathway databases) and to work with both paired and unpaired data.

For simplicity, we here only explored inter-layer links based on databases. However, such links could be improved in concrete biological applications considering inter-layer links derived from experimental evidence (e.g. resulting from ChIP-seq experiments instead of generalist motif databases). In addition, further developments of HuMMuS could allow to include additional single-cell data modalities, cell–cell interactions, and interactions from knowledge-based databases (e.g. REACTOME, GO). Finally, we here focused on community detection in GRNs to have a comparable output between HuMMuS and the current state-of-the-art. However, HuMMuS could further include in the future methods for community detection in HMLNs, thus allowing to detect cross-omics communities, providing a better picture of the complex interactions driving some biological processes.

## Supplementary Material

btae143_Supplementary_Data

## Data Availability

The code to run HuMMuS is available at https://github.com/cantinilab/HuMMuS together with tutorials. For the input data, all details to access them are reported in the second column of Supplementary Table S2 and links to access the preprocessed data are available at https://github.com/cantinilab/HuMMuS.
