# Supplementary material for: Molecular mechanisms reconstruction from single-cell multi-omics data with HuMMuS
Source: Bioinformatics. 2024 Mar 9;40(5):btae143. doi: 10.1093/bioinformatics/btae143 (PMC11065476; doi:10.1093/bioinformatics/btae143)
Supplement: btae143_Supplementary_Data [file btae143_supplementary_data.pdf]

Molecular mechanisms reconstruction from single-cell multi-omics data with HuMMuS

Remi Trimbour[1-2], Ina Maria Deutschmann[2], Laura Cantini[1-2]*

Supplementary Materials

Supplementary Text

HeterogeneoUs Multilayers for MUlti-omics Single-cell data (HuMMuS)

We developed HeterogeneoUs Multilayers for MUlti-omics Single-cell data (HuMMuS), a new tool for regulatory mechanisms inference from single-cell multi-omics data (https://github.com/cantinilab/HuMMuS).

HuMMuS is based on Heterogeneous Multilayer Networks (HMLNs). A HMLN is a network $M = (V_m, E_m, \mathbf{L}), m = 1, \ldots, M$, composed of M layers each of them containing different nodes $V_m$and different intra-layer links $E_m \subseteq V_m \times V_m$. Nodes of different layers are connected by inter-layers links encoded in **L**(Kivelä *et al.*, 2014; Baptista *et al.*, 2022). As summarized in Figure 1, we reconstruct HMLNs composed of three layers: The TF layer, containing unlinked TFs, the scATAC layer containing peak co-accessibility information inferred from scATAC data and the scRNA layer encoding transcriptional regulation inferred from scRNA data. Details on the layers construction are provided below.

Heterogeneous Multilayer Network (HMLN) construction

The standard structure we propose for molecular mechanisms reconstruction with HuMMuS is based on scRNA-seq and scATAC-seq data that does not need to be paired.

TF layer

TFs expressed in the scRNA data and having a known motif according to JASPAR or cisBP databases(Castro-Mondragon *et al.*, 2022; Weirauch *et al.*, 2014) were included in the TF layer. In the presented results, we did not include TF-TF interactions in the TF layer of HuMMuS, to make a fairer comparison with state-of-the-art methods. A second version of HuMMuS, called *HuMMuS + TF*, is also considered to test the added value brought by TF-TF links. In this case, TFs are linked based on post-translational interactions reported in OmniPath(Türei *et al.*, 2021).

scATAC layer

scATAC data are used in this layer to infer cis-regulatory interactions using Cicero(Pliner *et al.*, 2018). Cicero provides co-accessibility scores between peaks within given windows of the genome. We used 500kb as genomic window size for both human and mouse data, as done in(Kamimoto *et al.*, 2023; Pliner *et al.*, 2018). In addition, Cicero requires to define pseudocells, by averaging groups of N cells. In the following we used N=50, corresponding to the default Cicero value, with the only exception of the Liu dataset, where too few cells were present, thus requiring N=10. We then filtered the obtained network based on the co-accessibility scores: correlation threshold of zero for all datasets except the last dataset composed of three omics, where 0.2 is used. The obtained network is undirected and weighted.

scRNA layer

There are many methods to infer gene networks from scRNA data. Though it would be possible to use any network connecting genes without specifically regulatory hypotheses, we here chose to use GENIE3(Huynh-Thu *et al.*, 2010). GENIE3 is indeed one of the most popular methods to infer GRNs from RNA and scRNA data and it was shown to have better performances than other state-of-the-art tools in(Kang *et al.*, 2021; A *et al.*, 2020). Being the GENIE3 network a complete one, we filtered it keeping only the 10K links with the highest weight. Of note, the network obtained by GENIE3 is here considered as an undirected and weighted network thus allowing a random walk to move from a gene to all other genes co-regulated by a common TF.

TF-peak bipartite

To associate TFs to potential binding regions we used the function *AddMotifs* from the Signac package(Stuart *et al.*, 2021) and based on motifmatchr (Schep and University, 2023). This function can be, however, replaced by the users with others, if needed. TF binding-motifs were obtained from JASPAR and cisBP databases(Castro-Mondragon *et al.*, 2022; Weirauch *et al.*, 2014). JASPAR motifs were obtained through the JASPAR2020 R package(JASPAR2020). cisBP motifs already reformatted and deduplicated were accessed through chromVARmotifs R package(chromVARmotifs, 2023). To find overlap between TF binding motifs and scATAC-seq peak coordinates, elements were mapped on the genomic sequences from *BSgenome.Hsapiens.UCSC.hg38* and *BSgenome.Mmusculus.UCSC.mm10* for human and mouse, respectively. The obtained network is unweighted.

Peak-genes bipartite

We finally linked peaks to genes based on the distance of the peak from the transcription starting site (TSS) of the gene. We considered 500 bp before and after the TSS. We chose a small window since we wanted to directly link a gene to only potential promoters and leave the scATAC layer to give information on more distal regulatory regions, such as enhancers. The obtained network is unweighted.

For the computational time needed to reconstruct the Heterogeneous Multilayer Network (HMLN) with HuMMuS in a dataset of 55K cells scRNA and 9K cells scATAC see Supp Table 7. After the reconstruction of the HMLN random walk with restart has been used for mining its information.

Random walk with restart (RWR)

Random walk with restart (RWR) is a stochastic process consisting in a succession of steps from one node (i.e. the seed) to a neighboring one through the network's edges, with a probability to start again from the seed at each step. RWR can be used to explore HMLNs and to provide a measure of nodes' closeness across the layers, ensuring the existence of a unique stationary distribution(Kivelä *et al.*, 2014; Brin and Page, 1998). To run the RWR we here used MultiXrank, a python package proposing optimized RWR on universal multilayer networks(Baptista *et al.*, 2022).

The RWR of MultiXrank makes at every step three consecutive decisions: (1) it decides whether to restart from the seed or not; (2) it then decides on which layer to go based on different predefined probabilities; (3) it finally decides on which node to move, based on intra-layer links, if we stay in the same layer, and based on inter-links, if we move to another layer. We set the probability to restart from the seed and the probability to jump from one layer to another. The restart probability was set at 0.7 for all the results here presented, being the default value in MultiXrank and also used in other RWR applications(Baptista *et al.*, 2022; Didier *et al.*, 2015; Zhao *et al.*, 2015). Concerning the probability to jump from one layer to another, we set it to be equiprobable in all layers, including the starting one. This choice is aimed at having each omic contributing equally to the results. Of note, in the HuMMuS package, when possible, we parallelized RWRs to benefit from multi-core usage.

Possible outputs of HuMMuS

The final outputs of HuMMuS are: (i) the prediction of the targets of a Transcription Factor (TF), based on RWRs starting from each TF in the TF layer and exploring the full network until the scRNA layer; (ii) the prediction of the peaks bound by a given TF, based on RWRs starting from each TF in the TF layer and exploring the scATAC layer; (iii) the prediction of the regulatory regions (proximal and distal enhancers) associated to a given gene, based on RWRs starting in each gene of the scRNA layer and exploring the scATAC layer; (iv) the reconstruction of Gene Regulatory Networks (GRNs), based on RWRs starting in each gene of the scRNA layer and exploring the full network until the TF layer; (v) the extraction of communities in the GRN, reflecting tightly connected macromolecules in the HMLN frequently involved in the regulation of the same biological process or pathway(Barabási and Oltvai, 2004).

Benchmarking settings

Datasets and preprocessing

The benchmarking was realized on four datasets: Chen, Liu, Duren and Semrau (see Supp Table 2). The Chen and Liu datasets consisted of paired single-cell RNA sequencing (scRNA-seq) and single-cell chromatin accessibility profiling (scATAC-seq) data from human embryonic stem cells (hESCs). Duren and Semrau consisted of unpaired scRNA-seq data from mouse embryonic stem cells (mESCs). The Semrau dataset contained only scRNA-seq data, we thus used it together with the Duren's scATAC-seq data. Description of the data and download links can be found in Supp Table 2. Regarding data preprocessing, for both scRNA-seq and scATAC-seq data, we filtered out the features expressed in less than 1% of the cells. Gene counts were then log2-transformed and peak accessibilities were binarized by replacing the non-null values by 1.

Running the state-of-the-art methods

SCENIC+ (Bravo González-Blas *et al.*, 2023)

We first applied cisTopic, initializing a CistopicObject directly from the peak matrix since fragments files were not available for the four datasets. Topic modelling was realized with the *run_cgs_models* function and all default parameters. To handle data sparsity, accessibility imputation was also done according to SCENIC+ tutorials through the *impute_accessibility* function, with *scale_factor* = 1e6.

Since the benchmark was realized on single cell type datasets, we selected important regions for each topic using the Otsu method(Otsu, 1979) and by taking the 3000 top regions per topic. We then used pycisTarget with the precomputed motifs rankings and score per region, and motif annotation databases available at https://resources.aertslab.org/cistarget/databases/mus_musculus/mm10/screen/mc_v10_clust/region_based/ and https://resources.aertslab.org/cistarget/databases/homo_sapiens/hg38/screen/mc_v10_clust/region_based/ for mouse and human datasets respectively.

The search space around the gene was defined as 150kb upstream/downstream as suggested in the SCENIC+ tutorial, from the gene coordinates of the biomart_host matching each dataset. Finally, the functions *calculate_TFs_to_genes_relationships* *calculate_regions_to_genes_relationships*, *build_grn (min_target_genes=1, rho_threshold=0) format_egrns* were used successively with all default parameters, except those between parentheses. These two have been lowered to keep more regulations and test different thresholding in downstream evaluations.

Pando (Fleck *et al.*, 2022)

First, unpaired datasets were computationally paired with SCOTv2(Demetci *et al.*, 2022), running SCOTv2.align with default parameters (k=50, e=1e-3, balanced=True, rho=5e-2, normalize=True). Following the default Pando pipeline, pseudocells were then aggregated as described in https://github.com/quadbiolab/*organoid_regulomes/blob/main/pando/pseudocells.R* to reduce data sparsity(Fleck *et al.*, 2022). Motifs were obtained from JASPAR2020 and cisBP, and matched to

ATAC peaks with *find_motifs()*. The GRN network was finally inferred with *infer_grn()* using the parameters suggested in the Pando vignette, plus upstream = 100k, downstream = 100k and only_tss = TRUE to consider regulatory regions both downstream and upstream than the TSS, as done by the other tools here considered.

CellOracle (Kamimoto *et al.*, 2023)
We applied CellOracle as described in https://github.com/morris-lab/CellOracle. ScATAC-seq datasets were analyzed with Cicero to find co-accessible regions (co-accessibility score > 0.8) in a genomic window of 500kb. Peaks co-accessible with promoters were associated with genes through CellOracle *integrate_tss_peak_with_cicero* function. Peaks were also scanned with the CellOracle *TFinfo* function and its default parameters and default motifs to identify TF binding sites, to produce TF-gene edges. Finally, the TF-gene edges were inferred by *get_links* function with alpha = 10.

GENIE3(Huynh-Thu *et al.*, 2010)
The R implementation of GENIE3 has been considered here. For both human and mouse datasets, we used the TFs having a known motif in JASPAR2020 or cisBP and expressed in the scRNA-seq data.

TF targets predictions

The aim of this first benchmark is to test the ability of different methods to predict the targets of a Transcription Factor (TF). To do this prediction with HuMMuS, we set the TFs of interest as seeds of the RWR and explored the entire HMLN until the scRNA layer to find their target genes. The probabilities of the RWR have been set as follows: (i) for the default HuMMuS version, from the TF layer the only option was to move to the scATAC layer (as we have no link in the TF layer). We thus set a probability of 1 in the RWR to move from the TF layer to the scATAC layer. For *HuMMuS + TF*, we set a probability of 1/2 to stay in the TF layer and 1/2 to move to the scATAC layer; (ii) from the scATAC layer, we could stay on the layer or move either in the TF layer, either in the scRNA layer, we thus set the RWR probability to 1/3 to make all omics have the same relevance; (iii) from the scRNA layer, we could stay on the layer or move up into the scATAC layer we thus set the RWR probability to 1/2 to make all omics have the same relevance. The probability of restart was set to 0.7, default MultiXrank value. After RWR, we obtained, for each TF a ranking of putative target genes. The other state-of-the-art methods (CellOracle, GENIE3, Pando) provide a GRN, also corresponding to a list of TF-gene links reflecting a ranking of putative targets per TF. We thus evaluate performances comparing such rankings with ground-truth TF targets from(McCalla *et al.*, 2023) that are expressed in the scRNA data. The ground truth in(McCalla *et al.*, 2023) is composed of TF-target gene pairs for both hESCs and mESC obtained from the intersection of ChIP-seq data and perturbation experiments (impact of TFs KO/KD on gene expression).

For each method (HuMMuS, SCENIC+, CellOracle, GENIE3, Pando) and each TF in the ground-truth, we computed Fisher's exact tests and intersection sizes between the N top target genes and the ground-truth targets, with N varying in (3, 5, 10, 15, 20, 30, 40, 50, 75, 100). For each method, only TFs having at least 100 targets are considered. Finally, intersection performances are averaged across TFs, as TFs can vary from one method to another.

Regulatory regions identification

Predicting the peaks bounded by a TF

To predict the peaks bounded by each TF with HuMMuS, we focused on the TF layer and scATAC layer. RWRs were performed from each TF to explore the scATAC layer and find the peaks most close to them according to the RWR. The RWR probabilities were thus set to 1 for going from the TF layer to the scATAC layer (same argument for this as above); ½ to stay in the scATAC layer or move to the scRNA layer and 1 to go from the scRNA layer to the scATAC layer. The scRNA links are thus not used and the only scope of the scRNA layer is here to connect peaks associated to the regulation of the same gene. Once obtained a ranking of peaks for each TF, since the output of HuMMuS is a scoring of peaks and not a binary classification, we thresholded the ranking to only keep the top 100%, 80%, 60% or 20% of the ranking as our predictions. We then obtained Pando's TF-peak links from the GRN post regression. TF-peaks links in SCENIC+ were obtained from the pycisTarget predictions. Regarding CellOracle instead, TF-peak links were extracted from the backbone network, since it aggregates the peaks to calculate the TF-gene links. Since the backbone network of CellOracle is weighted according to Cicero, we further considered different Cicero thresholds (0.05, 0.2, 0.8). This list includes the default threshold of 0.8, plus additional lower thresholds since very few connections were kept with the default one. To then evaluate the quality of the obtained predictions, a ground-truth was defined from ReMap2022(Hammal *et al.*, 2022). We thus downloaded the list of the non-redundant peaks bound per TF computed in ReMap2022, using the 37 and 193 experiments available respectively from hESCs and mESCs. Only ReMap2022 peaks overlapping with the peaks of the scATAC data were considered as part of the ground-truth. Finally, we use F1 scores and proportion of true positives to compare the peaks rankings obtained from the SCENIC+, Pando, CellOracle and HuMMuS networks and the ground-truth peaks obtained from ReMap2022.

Predicting the regulatory regions (proximal and distal enhancers) associated to a gene

To predict the regulatory regions associated with a gene in HuMMuS, a RWR was computed starting from the gene as seed. No scRNA link was used, leading to a probability of 1 to go directly to the scATAC layer. Once reaching the scATAC layer, if no restart, the RWR remains in the scATAC layer with probability 1. This solution allows to explore the peaks associated with a gene based on the scATAC layer and thus potentially regulating the gene. Pando was not considered in this part of the benchmark since it does not infer peak-gene links independently from TF binding. In SCENIC+ peak-gene links were extracted after the regression model. To make the results of SCENIC+ and HuMMuS comparable, we took the same number of predicted enhancers for all the shared genes and filtered these predictions at different percentages (100%, 80%, 60%, 20%). In CellOracle, peak-gene links were extracted from the backbone networks and filtered according to correlation as suggested by the authors. As for TF-regions, we then considered different Cicero

thresholds: 0.05, 0.2 and 0.8, with 0.8 being the default value.

The obtained predictions were then compared with a ground-truth based on a combination of six enhancer databases. We first defined a list of potential enhancer-genes interactions from the union of PEGASUS(Clément *et al.*, 2020; Naville *et al.*, 2015), ENdb(Bai *et al.*, 2020) and EnhancerAtlas2.0(Gao and Qian, 2020). We then filtered this list, keeping only the links whose enhancers were present in the union of Fantom5(Forrest *et al.*, 2014), VISTA(Visel *et al.*, 2007), SCREEN.ENCODE(Moore *et al.*, 2020) databases. Finally, we only kept in the ground-truth enhancers overlapping with the peaks of the scATAC data. The quality of the overlap between predicted regulatory regions and the databases was finally assessed using F1 scores.

Community detection

As community detection methods well-suited for biological HMLN do not exist at the moment, we here compared community detection on the GRN output of HuMMuS vs. the GRNs obtained by the other methods. To obtain a GRN from HuMMuS we run, for each gene, a RWR starting from the gene as seed and arriving up to the TF layer to make TFs compete to regulate it. In the default HuMMuS version, we thus set the probabilities to ½ to stay in the scRNA layer or to jump from it to the scATAC layer, 1/3 to jump to any of the layers from the ATAC one, and a probability of 1 to reach the scATAC layer once reaching the TF layer. In *HuMMuS+TF,* we used the same RWR probabilities as above, except for the TF layer where we have a probability of ½ to stay in the layer and ½ to move back to the scATAC layer. Once obtained a GRN also for HuMMuS, we performed community detection on the GRNs of all methods (HuMMuS, SCENIC+, Pando, CellOracle and GENIE3). Only absolute weights were considered, all networks were filtered to the same density and community detection was finally realized with the Louvain clustering method(Blondel *et al.*, 2008) from the NetworkX implementation. To find the optimal clustering resolution for each of the methods, we tested 21 values between 0 to 2 with a step size of 0.1 (see Supp Table 5). Only resolutions providing at least 10 communities out of thousands of nodes (see Supp Table 4 for details on the number of nodes per method and dataset) were considered for the following part of the analysis. We considered five different databases to evaluate the quality of the clustering : GO Cellular Component, GO Biological Process, GO Molecular Function, KEGG 2021 (human) / 2019 (mouse) and Reactome 2016 (Kanehisa and Goto, 2000; Kanehisa *et al.*, 2023; Gillespie *et al.*, 2022; Ashburner *et al.*, 2000; Gene Ontology Consortium, 2021). For each method and resolution, we then used the enrichR package (Kuleshov *et al.*, 2016) to find enriched pathways in each of their communities. We then counted the number and the proportion of communities significantly enriched (p-value < 0.05 in the results presented Fig. 4) in at least one gene set of the database. For each method, we selected the resolution returning best performances.

HuMMuS applied to mouse cortex profiled for scRNA, scATAC and snmC

HuMMuS application from HMLN reconstruction to GRN extraction

To illustrate the potential of HuMMuS we used a single-cell dataset of cortical neurons composed of snmC, snATAC-seq and scRNA-seq. The data were downloaded from (Saunders *et al.*, 2018; atac_v1_adult_brain_fresh_5k - Datasets -Single Cell ATAC -Official 10x Genomics Support; Luo *et al.*, 2017). The snmC dataset was composed of 46,714 genes and 3386 cells; scRNA-seq was composed of 25,299 genes and 55,803 cells and scATAC-seq was composed of 155,093 peaks and 2317 cells. For scATAC and scRNA, we used preprocessed data in the h5ad files accessible at https://scglue.readthedocs.io/en/latest/data.html under the names *Saunders-2018* and *10x-Multiome-Pbmc10k*, while for snmC data, we used mCH methylation averaged per gene body (gene_level_mouse.txt) available at https://brainome.ucsd.edu/annoj/brain_single_nuclei/snmcSeq_processed_data.tar.gz and retained only the features expressed in more than 3% of the cells.

We then used HuMMuS to contract a HMLN consisting of four layers: a TF layer, a snmC layer, a scATAC layer, and a scRNA layer. To follow transcriptional regulation structure, we placed the snmC layer in the middle, connected with the scATAC layer and the scRNA layer. We did not link the snmC layer to the TF layer because TF binding motifs are specific to small regions, making gene bodies too large for precise binding motifs. As in the benchmark, we didn't put links in the TF layer. For the scATAC layer we used Cicero setting a co-accessibility score threshold at 0.2, as almost all correlations were above 0. The scRNA layer was computed with the python version of GRNBoost2, GENIE3 did not manage to get results on such a big dataset. Then the 50k links with the highest weights were kept. For the snmC layer, since we did not find methods designed to infer networks on methylation data, we used partial correlation from the pingouin0.5.3 python package, accessible at https://github.com/raphaelvallat/pingouin/tree/master. All the links with an absolute corrected correlation above 0.3 were kept. The inter-layer connections not involving the snmC layer were structured as in the benchmark. The connections between the snmC layer and the scATAC layer were set based on the distance of the scATAC peaks from the transcription start site (TSS) of the genes, nodes of the snmC layer (500 bp before and after the TSS). The connections between the snmC layer and the scRNA layer were just based on gene-gene correspondence.

After HMLN construction, using RWR from the gene layer up to the TF layer, we reconstructed a GRN. To give the same importance to each modality, the probability to go to any possible layer was the same. For the scATAC layer, we then have a probability of ¼ to go to each of the other layers or to stay in. For the scRNA layer and the snmC layer, we have a probability of ⅓ to stay in the layer, to move to the scATAC layer or to move to the other gene-node network. Finally, from the TFs layer it is only possible to jump to the scATAC layer.

Data analysis with the obtained GRN

Starting from the GRN provided by HuMMuS, we isolated regulons, corresponding to TFs and their linked genes, and

evaluated their activity in scRNA data using the unilinear model implemented in Decoupler (Badia-i-Mompel *et al.*, 2022). UMAP was then run on such an activity matrix to test the ability of the obtained regulons to cluster cells according to their cortical neuron sub-population of origin. Finally, TF activities were used to find top marker regulons of each cortical neuron sub-population focusing on the top 10 regulons per cortical sub-population.

References

A,P. et al. (2020) Benchmarking algorithms for gene regulatory network inference from single-cell transcriptomic data. Nature methods, 17.

Ashburner,M. et al. (2000) Gene Ontology: tool for the unification of biology. Nat Genet, 25, 25–29.

atac_v1_adult_brain_fresh_5k -Datasets -Single Cell ATAC - Official 10x Genomics Support.

Badia-i-Mompel,P. et al. (2022) decoupleR: ensemble of computational methods to infer biological activities from omics data. Bioinformatics Advances, 2, vbac016.

Bai,X. et al. (2020) ENdb: a manually curated database of experimentally supported enhancers for human and mouse. Nucleic Acids Res, 48, D51–D57.

Baptista,A. et al. (2022) Universal multilayer network exploration by random walk with restart. Commun Phys, 5, 1–9.

Barabási,A.-L. and Oltvai,Z.N. (2004) Network biology: understanding the cell's functional organization. Nat Rev Genet, 5, 101–113.

Blondel,V.D. et al. (2008) Fast unfolding of communities in large networks. J. Stat. Mech., 2008, P10008.

Bravo González-Blas,C. et al. (2023) SCENIC+: single-cell multiomic inference of enhancers and gene regulatory networks. Nat Methods, 20, 1355–1367.

Brin,S. and Page,L. (1998) The anatomy of a large-scale hypertextual Web search engine. Computer Networks and ISDN Systems, 30, 107–117.

Cao,Z.-J. and Gao,G. (2022) Multi-omics single-cell data integration and regulatory inference with graph-linked embedding. Nat Biotechnol, 40, 1458–1466.

Castro-Mondragon,J.A. et al. (2022) JASPAR 2022: the 9th release of the open-access database of transcription factor binding profiles. Nucleic Acids Research, 50, D165–D173.

chromVARmotifs (2023).

Clément,Y. et al. (2020) Enhancer–gene maps in the human and zebrafish genomes using evolutionary linkage conservation. Nucleic Acids Res, 48, 2357–2371.

Demetci,P. et al. (2022) SCOTv2: Single-Cell Multiomic Alignment with Disproportionate Cell-Type Representation. Journal of Computational Biology, 29, 1213–1228.

Didier,G. et al. (2015) Identifying communities from multiplex biological networks. PeerJ, 3, e1525.

Fleck,J.S. et al. (2022) Inferring and perturbing cell fate regulomes in human brain organoids. Nature, 1–8.

Forrest,A.R.R. et al. (2014) A promoter-level mammalian expression atlas. Nature, 507, 462–470.

Gao,T. and Qian,J. (2020) EnhancerAtlas 2.0: an updated resource with enhancer annotation in 586 tissue/cell types across nine species. Nucleic Acids Research, 48, D58–D64.

Gene Ontology Consortium (2021) The Gene Ontology resource: enriching a GOld mine. Nucleic Acids Res, 49, D325–D334.

Gillespie,M. et al. (2022) The reactome pathway knowledgebase 2022. Nucleic Acids Res, 50, D687–D692.

Hammal,F. et al. (2022) ReMap 2022: a database of Human, Mouse, Drosophila and Arabidopsis regulatory regions from an integrative analysis of DNA-binding sequencing experiments. Nucleic Acids Res, 50, D316–D325.

Huynh-Thu,V.A. et al. (2010) Inferring Regulatory Networks from Expression Data Using Tree-Based Methods. PLOS ONE, 5, e12776.

JASPAR2020 Bioconductor.

Kamimoto,K. et al. (2023) Dissecting cell identity via network inference and in silico gene perturbation. Nature, 614, 742–751.

Kanehisa,M. et al. (2023) KEGG for taxonomy-based analysis of pathways and genomes. Nucleic Acids Res, 51, D587–D592.

Kanehisa,M. and Goto,S. (2000) KEGG: kyoto encyclopedia of genes and genomes. Nucleic Acids Res, 28, 27–30.

Kang,Y. et al. (2021) Evaluating the Reproducibility of Single-Cell Gene Regulatory Network Inference Algorithms. Front Genet, 12, 617282.

Kivelä,M. et al. (2014) Multilayer networks. Journal of Complex Networks, 2, 203–271.

Kuleshov,M.V. et al. (2016) Enrichr: a comprehensive gene set enrichment analysis web server 2016 update. Nucleic Acids Res, 44, W90–W97.

Luo,C. et al. (2017) Single-cell methylomes identify neuronal subtypes and regulatory elements in mammalian cortex. Science, 357, 600–604.

McCalla,S.G. et al. (2023) Identifying strengths and weaknesses of methods for computational network inference from single-cell RNA-seq data. G3 (Bethesda), 13, jkad004.

Moore,J.E. et al. (2020) Expanded encyclopaedias of DNA elements in the human and mouse genomes. Nature, 583, 699–710.

Naville,M. et al. (2015) Long-range evolutionary constraints reveal cis-regulatory interactions on the human X chromosome. Nat Commun, 6, 6904.

Otsu,N. (1979) A Threshold Selection Method from Gray-Level Histograms. IEEE Transactions on Systems, Man, and Cybernetics, 9, 62–66.

Pliner,H.A. et al. (2018) Cicero predicts cis-regulatory DNA interactions from single cell chromatin accessibility data. Mol Cell, 71, 858-871.e8.

Saunders,A. et al. (2018) Molecular Diversity and Specializations among the Cells of the Adult Mouse Brain. Cell, 174, 1015-1030.e16.

Schep,A. and University,S. (2023) motifmatchr: Fast Motif Matching in R.

Stuart,T. et al. (2021) Single-cell chromatin state analysis with Signac. Nat Methods, 18, 1333–1341.

Türei,D. et al. (2021) Integrated intra- and intercellular signaling knowledge for multicellular omics analysis. Molecular Systems Biology, 17, e9923.

Visel,A. et al. (2007) VISTA Enhancer Browser--a database of tissue-specific human enhancers. Nucleic Acids Res, 35, D88-92.

Weirauch,M.T. et al. (2014) Determination and inference of eukaryotic transcription factor sequence specificity. Cell, 158, 1431–1443.

Zhao,Z.-Q. et al. (2015) Laplacian normalization and random walk on heterogeneous networks for disease-gene prioritization. Computational Biology and Chemistry, 57, 21–28.

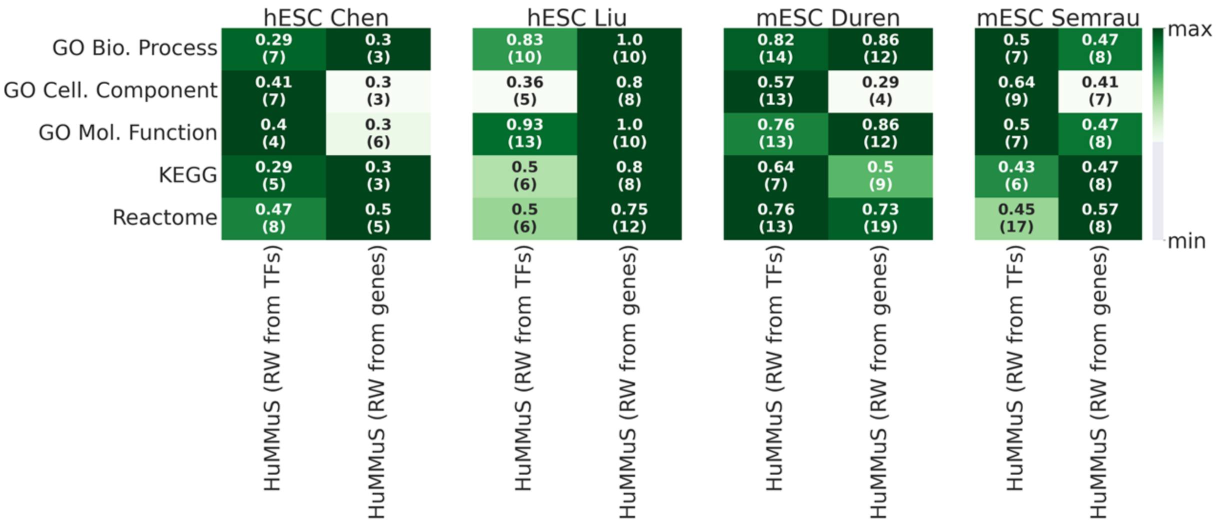

Supplementary Figure 1 - Enriched communities from different RWR exploration. (A) Heatmaps of percentage of enriched community when starting random walk with restart from the TFs and from the genes across the five biological databases. The values reported in the table correspond to the percentage of enriched communities, while those in parentheses are the actual number of enriched communities.

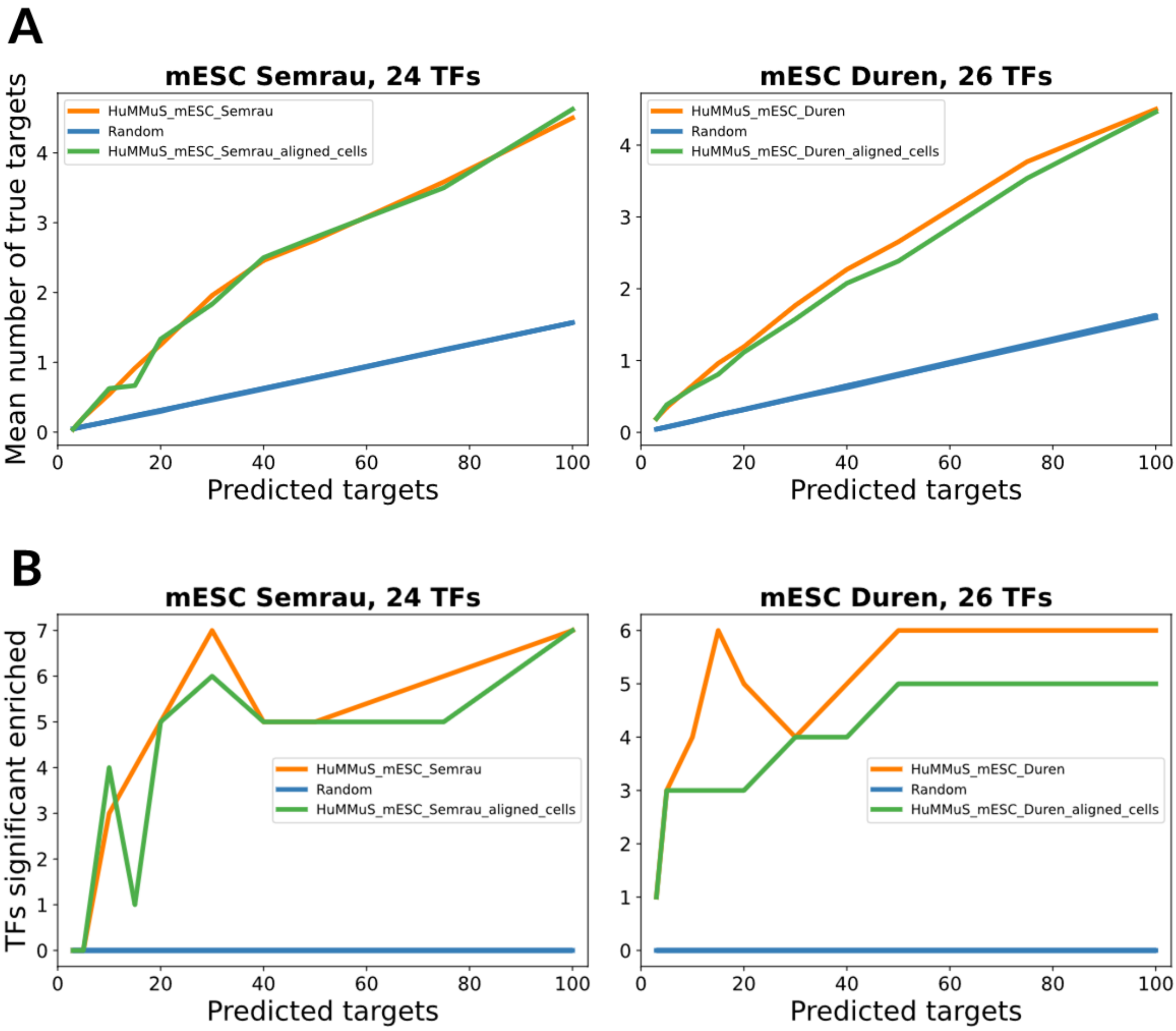

Supplementary Figure 2 - Transcription Factor (TF) - target genes prediction with and without cell pairing. (A) Average number of correctly predicted targets per TF. (B) Number of TFs with a significant number of correctly predicted targets (Fisher's exact test p-val<0.05). In (A-B) Colors correspond to different methods: orange (HuMMus on unpaired scATAC+scRNA-seq data), green (HuMMuS on paired scATAC+scRNA-seq data), blue (Random).

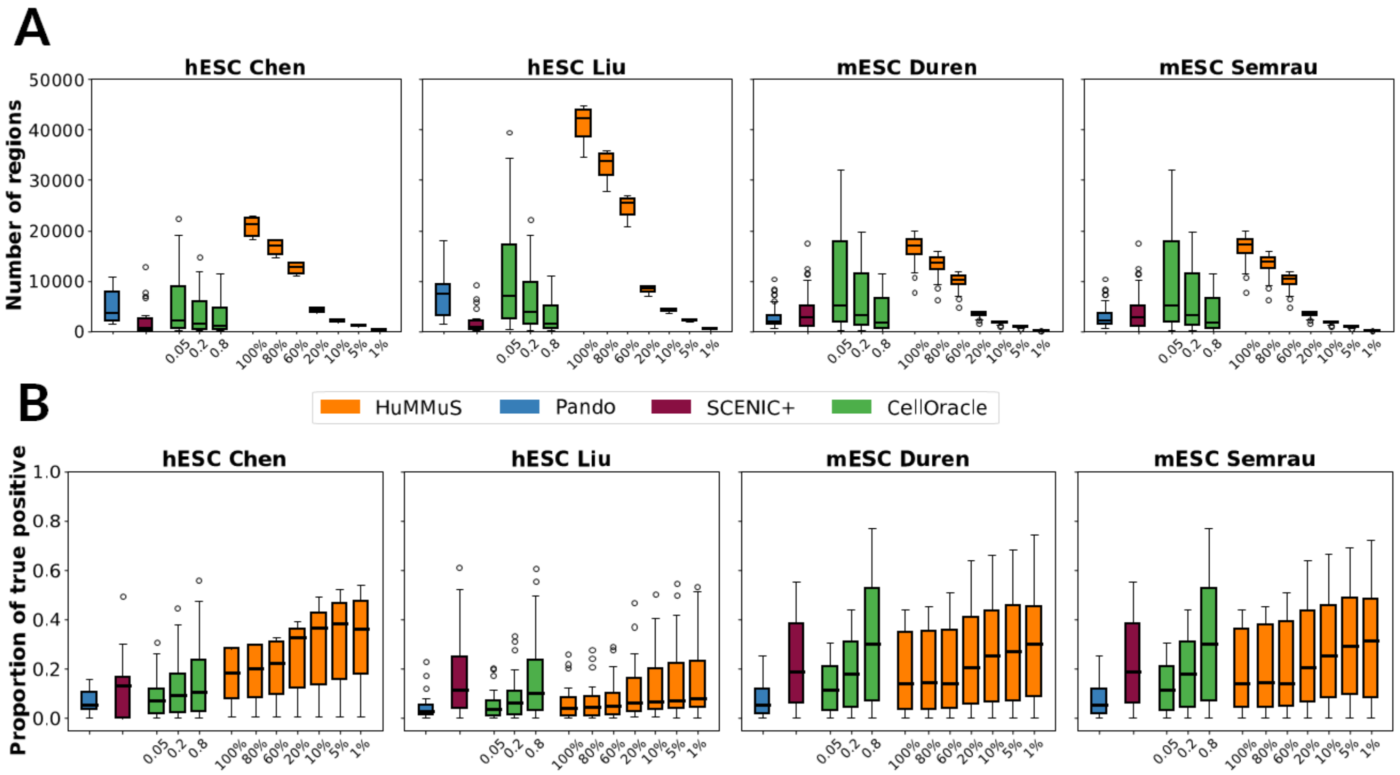

Supplementary Figure 3 - Number of regions and proportion of true positives detected per TF-region predictions' methods. (A) Distribution of the number of binding regions per TF inferred by Pando, CellOracle, SCENIC+ and HuMMuS; (B) Proportion of true positives in the predicted TF - binding regions pairs of the different methods. In (A-B) different colors correspond to different methods: HuMMuS (orange), Pando (blue), CellOracle (green), SCENIC+ (dark red).

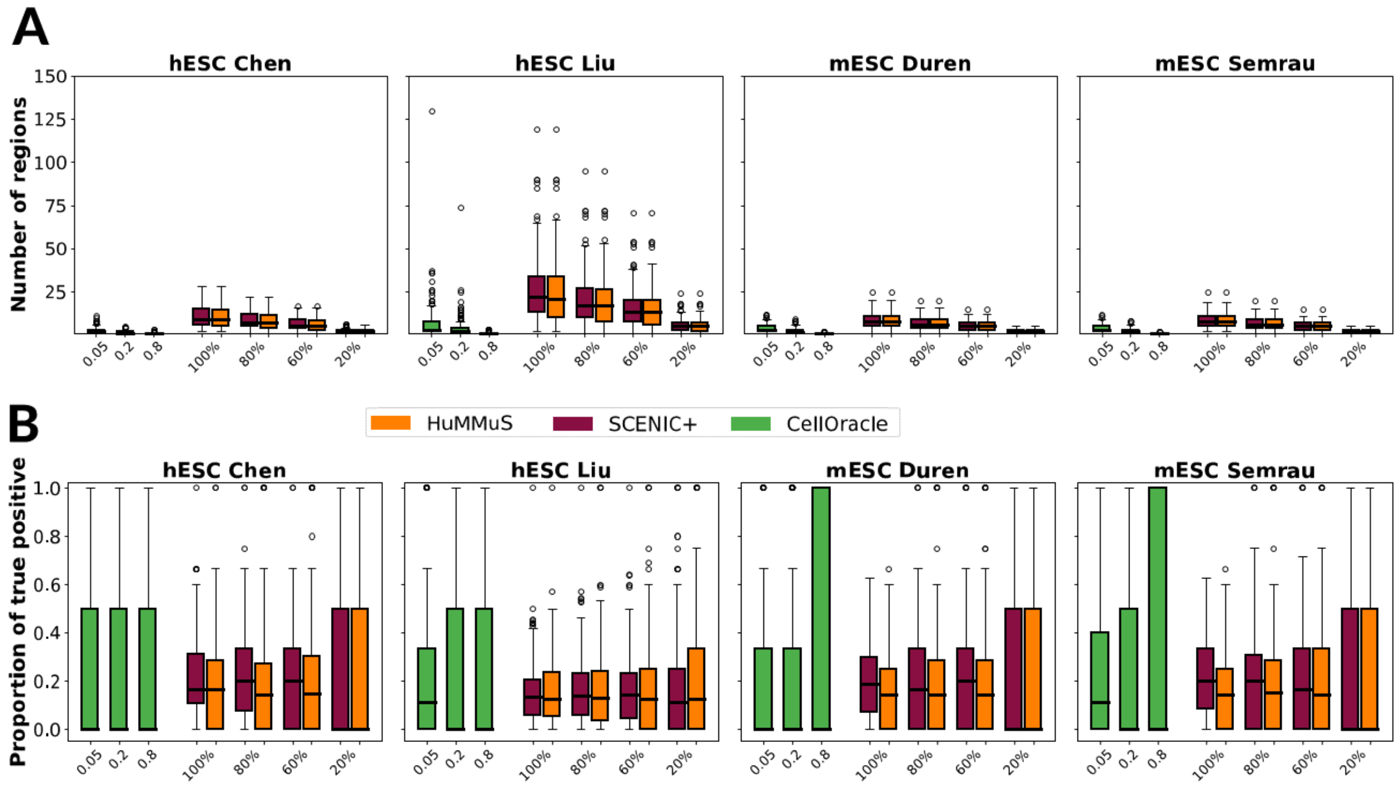

Supplementary Figure 4 - Number of regions and proportion of true positives detected per enhancer predictions' methods. (A) Distribution of the number of regulatory regions per gene inferred by CellOracle, SCENIC+ and HuMMuS. (B) Proportion of true positives in the predicted enhancers - gene pairs of the different methods. In (A, B) colors correspond to methods: HuMMuS (orange), CellOracle (green), SCENIC+ (dark red).

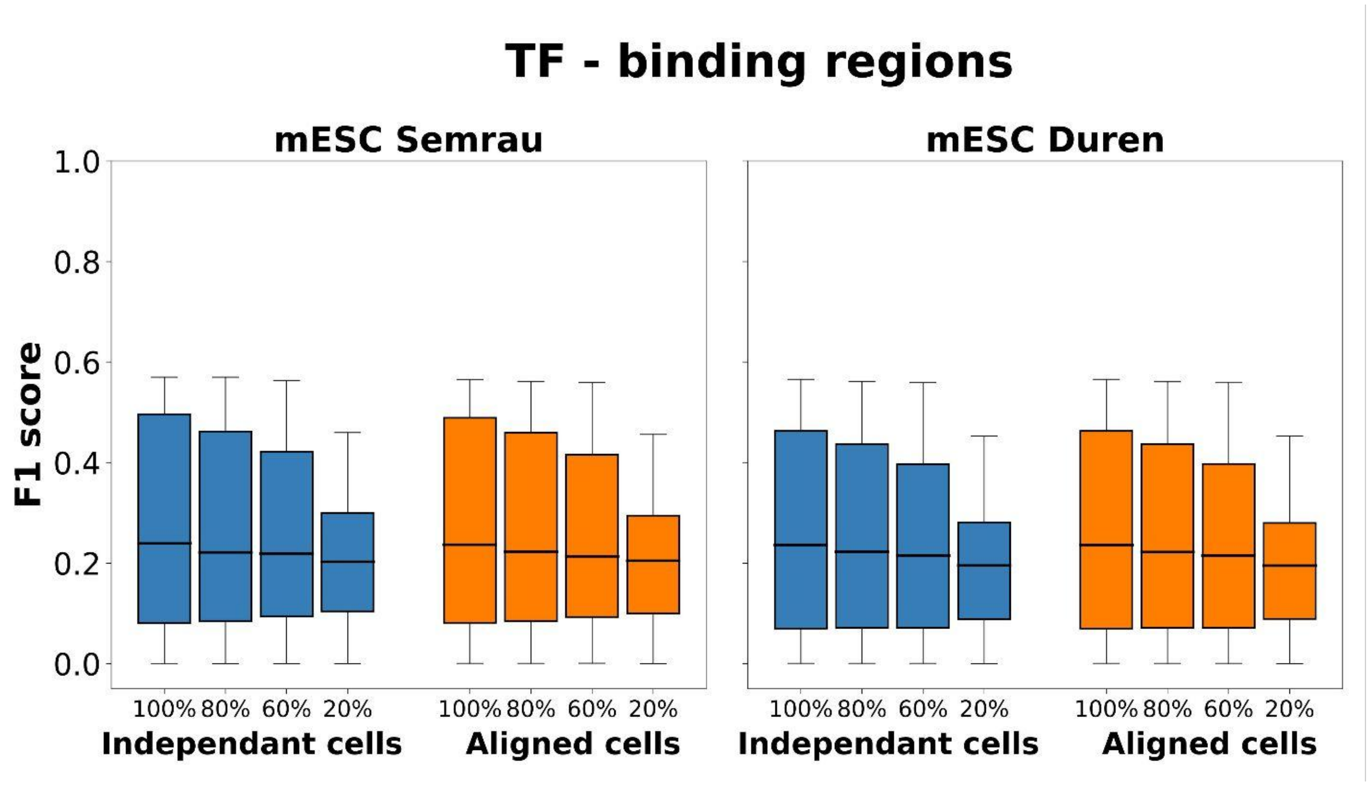

Supplementary Figure 5 - Binding regions and regulatory regions prediction with and without cell pairing. F1 score distributions of the intersection between the ground-truth of TF-peak associations and those inferred by HuMMuS. Different colors correspond to different data processing: orange (HuMMuS on unpaired scATAC and scRNA-seq data), blue (HuMMuS on paired scATAC and scRNA-seq data).

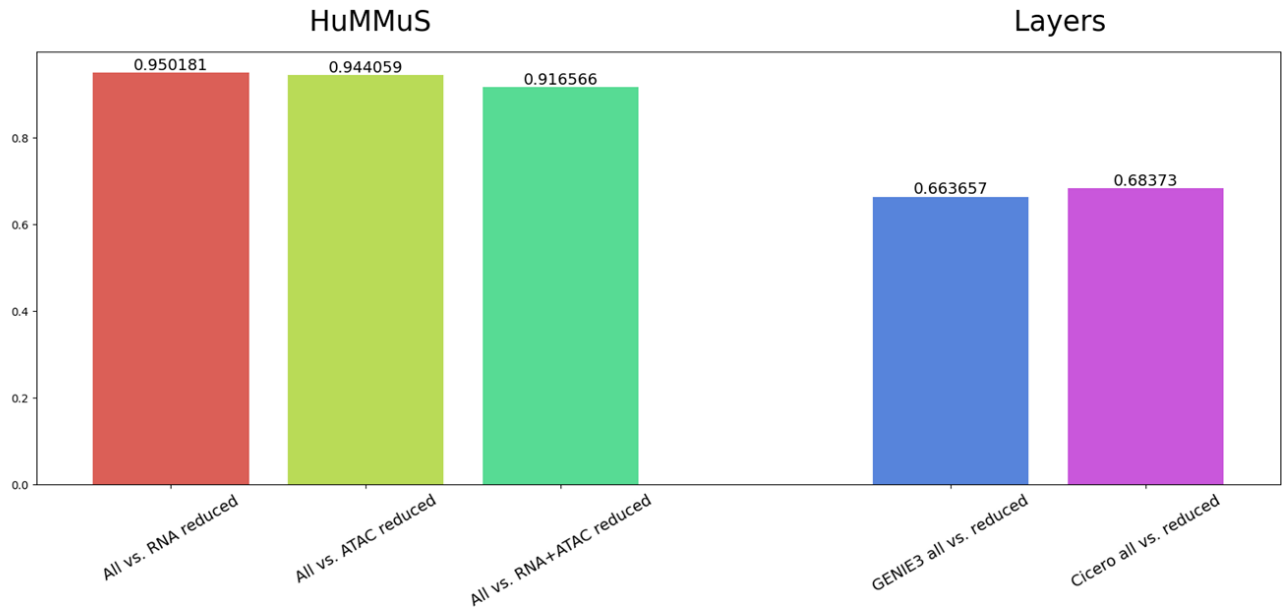

Supplementary Figure 6 - Spearman correlation between HuMMuS. Spearman correlation coefficient between GRNs inferred by HuMMuS on different subsets of a mouse cortex dataset (scRNA+scATAC-seq unpaired). Correlations have been computed between the complete dataset and 3 subsets of it, removing scRNA-seq cell and/or scATAC-seq measurements (barplots on the left). Correlations in between the corresponding scATAC layer and in between the corresponding scRNA layer obtained from these subsets have also been computed (barplots on the right). Three cortical neuron locations are present : MGE (184 cells), Layer 2/3 (614 cells) and Layer 6 (345 cells). In the reduced scRNA dataset, half of the Layer 2/3 neurons have been discarded from the scRNA-seq data. In the reduced scATAC dataset, half of the Layer 6 neurons have been discarded from the scATAC-seq data.

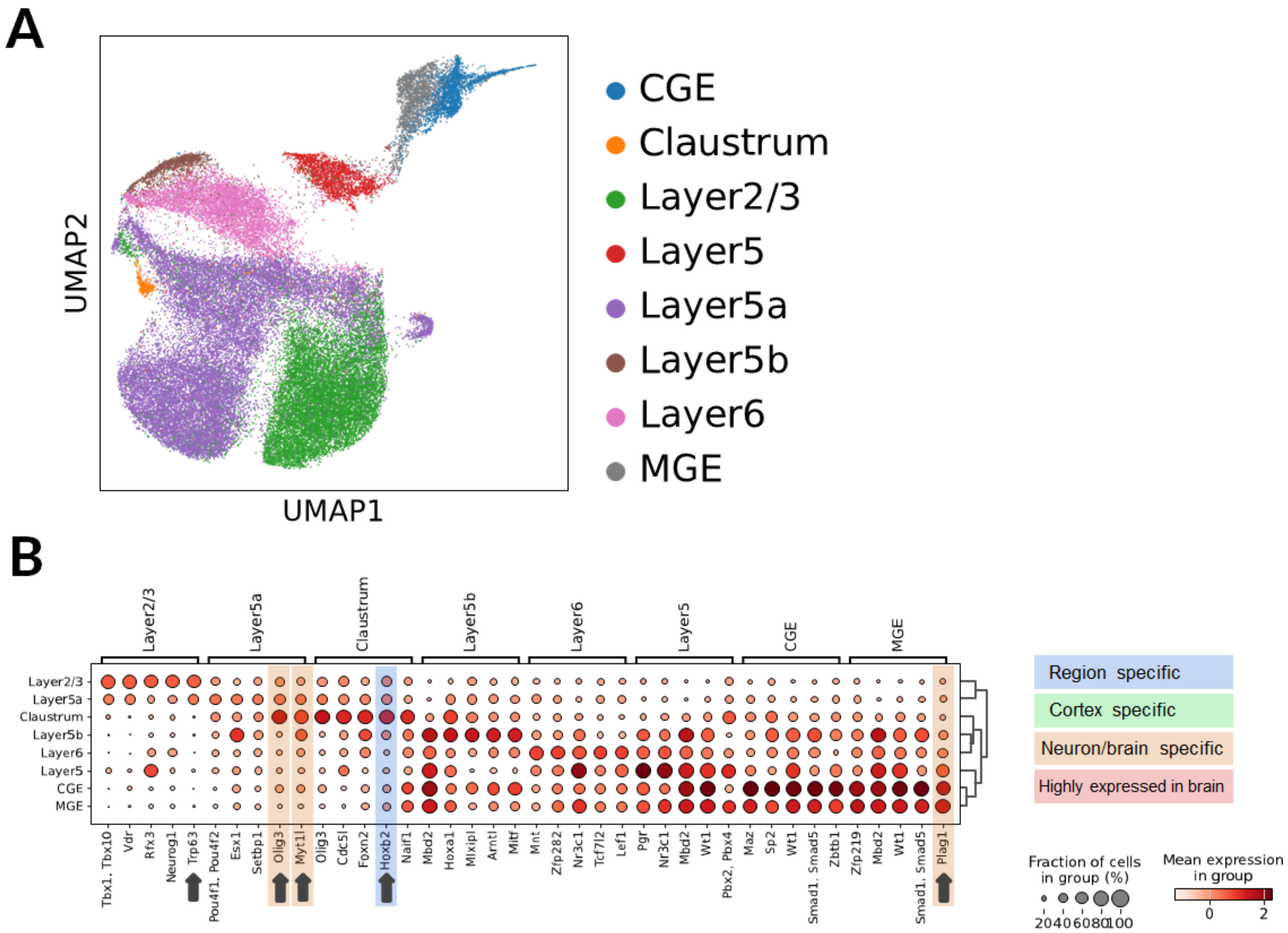

Supplementary Figure 7 - HuMMuS results on mouse cortex without methylation. (A) UMAP plot, in regard to Figure 5.B, showing the UMAP of the cells obtained from HuMMuS (without methylation layer) regulon activity. Cells are colored according to the label present in their original publication and in previous analyses. (B) Heatmap of activity for the top five TFs per cell population. Colors are used to denote the type of validation available; arrows indicate TFs lost once methylation is excluded from the analysis.

Supplementary Table 1. Spearman correlation between weighted edges of TF - target gene networks and GRNs, with/without using TF - TF interactions for each dataset. For each pair of networks, all common non-null edges were used.

		TF - target gene (no TF)	TF - target gene (TF)
Chen	GRN (no TF)	0.6287910691	
	GRN (TF)		0.6990991243
Liu	GRN (no TF)	0.7700942452	
	GRN (TF)		0.6732228101
Duren	GRN (no TF)	0.7168694993	
	GRN (TF)		0.6675759391
Semrau	GRN (no TF)	0.6189075068	
	GRN (TF)		0.6724702671

Supplementary Table 2. Collection of publicly data used in this study.(A) Description of the four datasets to benchmark HuMMuS in respect to state-of-the-art methods. It notably contains accessibility identifier/link and related originial publication. (B) List of the ground truth used to benchmark targets genes of transcription factors for the mESC and hESC datasets described above. (C) Databases of enhancers and regulatory regions combined to evaluate methods to retrieve regulatory regions important for each genes. (D) List of the databases used to evaluate enrichment of community detected from GRN benchmarking. (E) List and accessibility links to the data used for the 3 omics multilayer reconstruction. We used the table already preprocessed and formated by Cao et al., 2022.

A. Benchmark data: Gene expression and chromatin accessibility datasets

Dataset name	Data accession	Associated publication
hESC_Chen	NCBI (GSE126074):	•└(Chen et al., 2019)◘
scRNA	8595 features and 385 cells	Obtained by SNARE-seq. cell line mixture SNAREseq cDNA counts and chromatin counts, cell labels for filtering (ftp://ftp.ebi.ac.uk/ pub/ databases/mofa/snare_seq/cell_metadata.txt). Here, we only use H1 cells.
scATAC	36954 features and 385 cells	
hESC_Liu	https://github.com/hdsu-bioquant/scCAT/blob/master/data/HumanEmbryo/.	•└(Liu et al., 2019)◘
scRNA	23153 features and 72 cells	Obtained by scCAT. Human embryo RNA-seq counts, ATAC-seq counts, and annotation data downloaded from github link
scATAC	68952 features and 72 cells	
mESC_Duren	NCBI (GSE115968): scRNA-seq_RA_D4, and NCBI (GSE115970): scATAC-seq_RA_D4	•└(Duren et al., 2018; Zeng et al., 2019)◘
scRNA	15299 features and 464 cells	415 scATAC-seq samples generated for the retinoic acid-induced mESC differenation at day 4.
scATAC	23176 features and 414 cells	464 scRNA-seq samples generated for the retinoic acid-induced mESC differenation at day 4.
mESC_Semrau	NCBI (GSM2098553) part of (GSE79578): scrbseq_96h (scRNA-seq), but no scATAC-seq. We use the scATAC-seq from mESC_Duren, NCBI (GSE115970).	•└(Semrau et al., 2017)◘
scRNA	10243 features and 384 cells	scRNA seq data generated alone. scATAC-seq data of Semrau et al. has been used as peak layer

B. Ground truth table of target genes of transcription factors

Dataset name	Data accession link	Associated publication
mESC & hESC TF GT	https://zenodo.org/record/5909090/files/gold_standard_datasets.zip?download=1, Tables used hESC_chipunion_KDUnion_intersect.txt, and mESC_chipunion_KDUnion_intersect.txt, downloaded: 6th April 2022	•└(Stone et al., 2022)◘

C. Databases used to construct Ground truth table of enhancers for genes

Databases name	Data accession	Associated publication
EnhancerAtlas2.0	http://www.enhanceratlas.org/indexv2.php, Table used (ESC_neuron_EP.txt, ESC_Bruce4_EP.txt, ESC_J1_EP.txt, and ESC_KH2_EP.txt): enhancer-gene interactions - Homo sapiens (hg19: ESC_neuron) and Mus musculus (mm9: ESC_Bruce4, ESC_J1, ESC_KH2), Downloaded: 4th April 2022, Preprocessing: table formatting, convert hg19 to hg38, mm9 to mm10, and ENSEMBL Gene IDs to Gene Symbol	•└(Gao & Qian, 2020)◘
ENdb	http://www.licpathway.net/ENdb/, Table used (ENdb_enhancer.txt): All the experimentally confirmed enhancers (hg19 and mm10), Downloaded: 4th April 2022, Preprocessing: convert hg19 to hg38	•└(Bai et al., 2020)◘
SCREEN (ENCODE)	http://screen.encodeproject.org, Table used (GRCh38-cCREs.bed.html and mm10-ccREs.bed.html): all human cCREs (hg38) and all mouse cCREs (mm10), Downloaded: 4th April 2022, Preprocessing: none	•└(The ENCODE Project Consortium et al., 2020)◘
VISTA	http://enhancer.lbl.gov, Table used (imagedb3.pl.html): all 3281 elements (hg19 and mm9), Downloaded: 4th April 2022, Preprocessing: delete sequences, bring into table form, convert hg19 to hg38, mm9 to mm10	•└(Visel et al., 2007)◘
PEGASUS	ftp://ftp.biologie.ens.fr/pub/dyogen/PEGASUS/, Table used (hg19_CNEs_PEGASUS.data.gz): PEGASUS predictions for the human genome (hg19), Downloaded: 4th April 2022, Preprocessing: convert hg19 to hg38, hg19 to mm10, and ENSEMBL Gene IDs to Gene Symbol	•└(Naville et al., 2015; Clément et al., 2020)◘
Fantom5	https://slidebase.binf.ku.dk/human_enhancers/presets, Table used (hg19_enhancer_promoter_correlations_distances_cell_type.txt.gz): Enhancer-Promoter Cell Type Associations in 5.Enhancer - FANTOM Robust Promoter associations, Downloaded: 12th May 2022, Preprocessing: convert hg19 to hg38, hg19 to mm10 for enhancer and also promoter regions	•└(Forrest et al., 2014)◘

D. Databases for gene enrichment analyses included in enrichR •└(Chen et al., 2013; Kuleshov et al., 2016; Xie et al., 2021)◘

Databases name	Data accession	Associated publication
Gene Ontology	GO_Biological_Processes_2021, GO_Cellular_Component_2021, and GO_Molecular_Function_2021	•└(Ashburner et al., 2000; Gene Ontology Consortium, 2021)◘
Kyoto Encyclopedia of Genes and Genomes	KEGG_2021_Human and KEGG_2019_Mouse	•└(Kanehisa & Goto, 2000; Kanehisa, 2019; Kanehisa et al., 2021)◘
Reactome	Reactome_2016 (citation refers to latest Reactome version not used within enrichR)	•└(Gillespie et al., 2022)◘

E. Dataset to test inclusion of 4th layer: Gene expression, chromatin accessibility, and HiC data

Dataset name	Data accession	Associated publication
scRNA mouse cortex	http://download.gao-lab.org/GLUE/dataset/Saunders-2018.h5ad	Saunders et al., 2018
scATAC mouse cortex	http://download.gao-lab.org/GLUE/dataset/10x-ATAC-Brain5k.h5ad	https://support.10xgenomics.com/single-cell-atac/datasets/1.1.0/atac_v1_adult_brain_fresh_5k
snmC mouse cortex	http://download.gao-lab.org/GLUE/dataset/Luo-2017.h5ad	Luo et al., 2017

Supplementary Table 3. General description of the different multilayers components. This table contains number of nodes and edges in each component of the multilayers analysed in this article

	Layer / Bipartite	3 omics mouse cortex	hESC Chen	hESC Liu	mESC Duren	mESC Semrau
**TF layer (standard HuMMuS)**	Number of nodes	717	220	670	607	334
**TF layer (from OmniPath)**	Number of nodes		432	432	364	364
	Number of edges		1403	1403	1046	1046
**scATAC layer**	Number of nodes	82108	25102	48207	20779	20779
	Number of edges	302651	96104	439628	72986	72986
**scRNA layer**	Number of nodes	9349	5095	5712	4520	5695
	Number of edges	50000	10000	10000	10000	10000
**snmC layer**	Number of nodes	5494				
	Number of edges	558845				
**TF --> scATAC bipartite**	Number of sources	717	220	670	607	334
	Number of targets	153507	25102	48207	20779	20779
	Number of edges	10685078	619902	3520936	1373435	792771
**scATAC--> scRNA bipartite**	Number of sources	36494	4989	3624	3036	4772
	Number of targets	16214	3780	2086	2661	4367
	Number of edges	36494	5230	3690	3096	4986
**scATAC --> snmC bipartite**	Number of sources	35210				
	Number of targets	17284				
	Number of edges	38299				
**snmC --> scRNA bipartite**	Number of sources	24504				
	Number of targets	24504				
	Number of edges	24504				

Supplementary Table 4. Density comparison between the GRNs of the different methods. Table containing the number of TFs, genes, edges and the density of the GRNs defined by each method for the benchmark.

		Number of TFs	Number of genes	Number of edges	Density
**hESC_Chen**	**CellOracle**	252	5925	369196	0.24731
	**HuMMuS**	220	5095	1120680	1
	**HuMMuS + TF**	426	5383	2170332	0.94661
	**SCENIC+**	536	8595	652237	0.14159
	**Pando**	220	8152	270817	0.15102
	**GENIE3**	220	8595	1875712	0.99208
**hESC_Liu**	**CellOracle**	716	9749	1390371	0.19921
	**HuMMuS**	670	5733	3801638	0.9899
	**HuMMuS + TF**	432	5675	2451600	0.98374
	**SCENIC+**	1387	23153	2884201	0.08977
	**Pando**	517	15517	232767	0.02902
	**GENIE3**	670	23153	12424093	0.80094
**mESC_Semrau**	**CellOracle**	388	7662	706606	0.23772
	**HuMMuS**	334	5695	1901796	1
	**HuMMuS + TF**	360	5695	2050200	0.97109
	**SCENIC+**	738	10243	1157290	0.15311
	**Pando**	334	9658	695894	0.21575
	**GENIE3**	334	10243	3300296	0.96477
**mESC_Duren**	**CellOracle**	664	10473	1337231	0.19231
	**HuMMuS**	607	4570	2741871	0.98864
	**HuMMuS + TF**	364	4601	1644271	0.98201
	**SCENIC+**	1207	15299	2065227	0.11185
	**Pando**	602	14045	1240683	0.14675
	**GENIE3**	607	15299	8777184	0.94522

Supplementary Table 5. Number of significantly enriched communities (p value < 0.05) detected for each method, resolution, and database used.

			Resolution																				
			0	0.1	0.2	0.3	0.4	0.5	0.6	0.7	0.8	0.9	1	1.1	1.2	1.3	1.4	1.5	1.6	1.7	1.8	1.9	2
**hESC_Chen**	**CellOracle**	GO_Biological_Process_2021	1	1	1	1	1	1	1	1	2	3	7	10	9	12	18	19	27	33	29	35	38
		GO_Cellular_Component_2021	1	1	1	1	1	1	1	1	2	3	10	17	14	21	16	22	25	24	28	27	36
		GO_Molecular_Function_2021	1	1	1	1	1	1	1	1	2	3	10	16	15	17	17	21	24	33	36	37	36
		KEGG_2021_Human	1	1	1	1	1	1	1	1	2	3	5	4	5	10	8	11	10	11	13	12	10
		Reactome_2016	1	1	1	1	1	1	1	1	2	3	10	14	11	14	17	20	21	23	20	29	32
	**Pando**	GO_Biological_Process_2021	1	2	2	2	2	2	2	2	2	2	2	3	4	3	4	4	2	2	2	3	3
		GO_Cellular_Component_2021	1	2	2	2	2	2	2	2	2	2	2	2	3	2	3	2	1	1	1	2	2
		GO_Molecular_Function_2021	1	2	2	2	2	2	2	2	2	2	2	3	4	3	4	4	2	2	2	3	3
		KEGG_2021_Human	1	2	2	2	2	2	2	2	2	2	2	1	3	2	3	3	1	1	1	2	2
		Reactome_2016	1	2	2	2	2	2	2	2	2	2	2	2	4	3	4	4	2	2	2	3	3
	**GENIE3**	GO_Biological_Process_2021	1	1	1	1	1	1	1	1	1	3	7	8	13	15	12	17	17	15	15	20	23
		GO_Cellular_Component_2021	1	1	1	1	1	1	1	1	1	5	13	14	18	18	20	21	20	20	22	20	19
		GO_Molecular_Function_2021	1	1	1	1	1	1	1	1	1	6	6	10	12	18	15	23	21	21	22	25	27
		KEGG_2021_Human	1	1	1	1	1	1	1	1	1	4	4	5	7	6	13	9	10	11	13	10	12
		Reactome_2016	1	1	1	1	1	1	1	1	1	7	10	11	10	10	11	14	17	16	14	20	19
	**HuMMuS + TF**	GO_Biological_Process_2021	1	1	1	1	1	1	1	1	1	3	4	9	9	11	12	18	22	24	21	23	26
		GO_Cellular_Component_2021	1	1	1	1	1	1	1	1	1	3	5	5	11	8	8	9	11	16	15	14	14
		GO_Molecular_Function_2021	1	1	1	1	1	1	1	1	1	3	7	11	10	13	13	16	16	15	20	15	22
		KEGG_2021_Human	1	1	1	1	1	1	1	1	1	2	5	6	8	7	6	9	10	10	11	8	12
		Reactome_2016	1	1	1	1	1	1	1	1	1	4	5	8	12	15	19	20	22	26	28	21	31
	**HuMMuS**	GO_Biological_Process_2021	1	1	1	1	1	1	1	1	1	2	4	7	10	7	15	12	18	14	27	24	27
		GO_Cellular_Component_2021	1	1	1	1	1	1	1	1	1	3	4	5	5	7	9	14	13	18	18	20	22
		GO_Molecular_Function_2021	1	1	1	1	1	1	1	1	1	2	5	6	4	9	14	9	12	12	13	20	24
		KEGG_2021_Human	1	1	1	1	1	1	1	1	1	2	5	5	7	8	10	12	8	14	15	15	18
		Reactome_2016	1	1	1	1	1	1	1	1	1	3	5	5	5	8	13	17	12	21	19	21	30
	**SCENIC+**	GO_Biological_Process_2021	1	1	1	1	1	1	1	1	1	6	7	11	13	9	18	18	16	18	20	24	20
		GO_Cellular_Component_2021	1	1	1	1	1	1	1	1	1	11	12	14	17	18	18	19	22	23	24	23	25
		GO_Molecular_Function_2021	1	1	1	1	1	1	1	1	2	9	12	13	12	12	20	22	21	22	25	22	22
		KEGG_2021_Human	1	1	1	1	1	1	1	1	1	3	5	6	8	7	7	8	6	10	13	9	11
		Reactome_2016	1	1	1	1	1	1	1	1	2	9	12	16	14	19	17	20	19	21	22	23	24
**hESC_Liu**	**CellOracle**	GO_Biological_Process_2021	1	1	1	1	1	1	2	2	3	5	6	11	15	11	14	22	24	20	27	27	38
		GO_Cellular_Component_2021	1	1	1	1	1	1	2	2	3	5	6	9	13	15	16	17	22	24	21	27	27
		GO_Molecular_Function_2021	1	1	1	1	1	1	2	2	3	5	6	10	14	18	23	20	27	29	34	30	33
		KEGG_2021_Human	1	1	1	1	1	1	2	2	3	4	5	8	10	11	11	11	13	12	15	14	25
		Reactome_2016	1	1	1	1	1	1	2	2	3	4	5	8	8	13	9	13	16	17	20	23	29
	**Pando**	GO_Biological_Process_2021	1	1	1	1	1	0	1	2	1	2	2	2	2	4	3	3	1	3	3	4	4
		GO_Cellular_Component_2021	1	1	1	1	1	2	1	1	1	1	1	1	3	3	3	3	3	3	3	2	2
		GO_Molecular_Function_2021	1	1	1	1	1	1	2	2	2	2	2	2	2	3	3	2	2	1	2	2	1
		KEGG_2021_Human	1	1	1	1	1	1	0	1	1	2	1	2	2	2	2	3	2	2	2	1	2
		Reactome_2016	1	1	1	2	1	3	1	2	2	3	2	2	3	2	5	4	3	3	4	4	4
	**GENIE3**	GO_Biological_Process_2021	0	0	0	1	1	1	1	1	1	1	2	2	2	3	2	2	2	3	2	3	2
		GO_Cellular_Component_2021	0	0	0	1	1	1	1	1	1	1	2	2	2	3	2	2	3	2	2	2	2
		GO_Molecular_Function_2021	0	0	0	1	1	1	1	1	1	1	2	3	2	2	1	2	2	3	2	3	3
		KEGG_2021_Human	0	0	0	1	1	1	1	1	1	2	2	3	3	3	3	3	3	3	3	3	3
		Reactome_2016	0	0	0	1	1	1	1	1	2	2	2	2	2	3	2	2	3	2	2	3	3
	**HuMMuS + TF**	GO_Biological_Process_2021	1	1	1	1	1	1	1	2	3	5	9	12	15	19	21	25	26	34	35	40	43
		GO_Molecular_Function_2021	1	1	1	1	1	1	1	2	3	4	5	7	10	10	11	6	7	10	12	14	20
		KEGG_2021_Human	1	1	1	1	1	1	1	2	3	5	9	12	16	18	22	25	28	35	38	41	42
		Reactome_2016	1	1	1	1	1	1	1	2	3	5	5	8	10	11	8	11	16	14	17	19	18
		GO_Cellular_Component_2021	1	1	1	1	1	1	1	2	3	5	8	10	12	15	15	20	20	25	26	25	29
	**HuMMuS**	GO_Biological_Process_2021	1	1	1	1	1	1	1	1	2	5	7	11	16	21	22	24	30	41	42	50	47
		GO_Cellular_Component_2021	1	1	1	1	1	1	1	1	2	3	5	6	10	9	9	8	13	10	19	20	19
		GO_Molecular_Function_2021	1	1	1	1	1	1	1	1	2	5	7	11	17	23	24	31	34	42	48	53	54
		KEGG_2021_Human	1	1	1	1	1	1	1	1	2	4	5	5	9	8	8	10	12	19	18	18	24
		Reactome_2016	1	1	1	1	1	1	1	1	2	3	6	8	12	12	15	17	20	24	27	33	33
	**SCENIC+**	GO_Biological_Process_2021	0	0	0	0	1	2	1	1	3	2	3	5	5	5	5	5	5	4	4	4	4
		GO_Cellular_Component_2021	0	0	0	0	0	2	2	1	1	2	3	4	5	4	4	6	4	6	5	7	5
		GO_Molecular_Function_2021	0	0	0	0	0	1	1	1	1	2	3	3	3	3	3	4	4	5	4	6	4
		KEGG_2021_Human	0	0	0	0	0	1	1	1	1	1	1	4	3	3	3	3	3	3	3	4	3
		Reactome_2016	0	0	0	0	0	2	1	1	2	3	3	4	4	4	4	5	4	6	5	6	6
**mESC_Duren**	**CellOracle**	GO_Biological_Process_2021	1	1	1	1	1	1	1	1	2	4	4	12	15	24	27	33	37	41	42	46	52
		GO_Cellular_Component_2021	1	1	1	1	1	1	1	1	2	5	4	10	10	17	21	28	26	33	33	34	36
		GO_Molecular_Function_2021	1	1	1	1	1	1	1	1	2	4	4	10	12	25	23	22	31	35	33	40	41
		KEGG_2019_Mouse	1	1	1	1	1	1	1	1	2	5	4	8	9	16	20	17	19	21	23	25	34
		Reactome_2016	1	1	1	1	1	1	1	1	2	5	5	10	17	21	18	27	31	30	35	45	45
	**Pando**	GO_Biological_Process_2021	1	1	2	2	2	2	2	2	2	2	2	9	7	9	11	18	15	11	21	19	28
		GO_Cellular_Component_2021	1	1	2	2	2	2	2	2	2	2	2	7	4	6	9	12	14	13	17	12	21
		GO_Molecular_Function_2021	1	2	2	2	2	2	2	2	2	2	2	9	8	8	11	16	17	17	18	18	24
		KEGG_2019_Mouse	1	2	2	1	1	2	2	2	2	2	2	6	5	5	6	7	13	12	13	12	14
		Reactome_2016	1	2	2	2	1	2	2	2	2	2	2	9	5	6	11	9	15	13	16	14	19
	**GENIE3**	GO_Biological_Process_2021	1	1	1	1	1	1	1	1	2	3	2	4	8	9	15	4	13	13	11	16	16
		GO_Cellular_Component_2021	1	1	1	1	1	1	1	1	2	2	2	3	5	10	9	9	12	12	18	21	20
		GO_Molecular_Function_2021	1	1	1	1	1	1	1	1	1	3	3	1	3	8	11	9	16	10	13	18	14
		KEGG_2019_Mouse	1	1	1	1	1	1	1	1	1	3	3	2	3	5	7	5	11	9	9	13	10
		Reactome_2016	1	1	1	1	1	1	1	1	2	3	3	2	6	5	8	10	10	11	15	20	21
		GO_Biological_Process_2021	1	1	1	1	1	1	1	1	2	4	8	10	12	11	14	19	15	23	23	25	29
		GO_Cellular_Component_2021	1	1	1	1	1	1	1	1	2	4	7	9	7	9	10	15	9	16	15	13	16

	**HuMMuS + TF**	GO_Molecular_Function_2021	1	1	1	1	1	1	1	1	2	4	7	8	8	13	16	14	20	25	20	23	34
		KEGG_2019_Mouse	1	1	1	1	1	1	1	1	2	4	5	8	8	12	11	15	14	15	15	18	21
		Reactome_2016	1	1	1	1	1	1	1	1	2	4	10	10	11	13	17	22	17	20	20	23	26
	**HuMMuS**	GO_Biological_Process_2021	1	1	1	1	1	1	1	1	2	3	8	9	8	11	16	26	29	30	33	38	51
		GO_Cellular_Component_2021	1	1	1	1	1	1	1	1	2	3	7	7	6	9	10	13	12	18	22	21	25
		GO_Molecular_Function_2021	1	1	1	1	1	1	1	1	2	3	6	6	9	15	12	15	22	28	30	26	34
		KEGG_2021_Human	1	1	1	1	1	1	1	1	2	3	4	7	7	9	13	13	16	22	23	19	19
		Reactome_2016	1	1	1	1	1	1	1	1	2	3	9	8	11	13	17	20	25	28	28	27	33
	**SCENIC+**	GO_Biological_Process_2021	1	1	1	1	1	1	1	1	2	5	4	5	5	10	7	9	13	13	10	18	19
		GO_Cellular_Component_2021	1	1	1	1	1	1	1	1	2	3	2	4	7	9	7	9	13	12	14	16	14
		GO_Molecular_Function_2021	1	1	1	1	1	1	1	1	2	3	1	3	4	5	4	8	9	11	16	18	17
		KEGG_2021_Human	1	1	1	1	1	1	1	1	2	3	2	2	4	6	2	6	8	7	9	12	14
		Reactome_2016	1	1	1	1	1	1	1	1	2	4	5	5	6	8	7	10	17	12	14	19	21
**mESC_Semrau**	**CellOracle**	GO_Biological_Process_2021	1	1	1	1	1	1	1	1	2	3	7	10	21	27	36	41	41	48	55	67	73
		GO_Cellular_Component_2021	1	1	1	1	1	1	1	1	2	3	7	11	13	11	22	24	29	33	34	38	44
		GO_Molecular_Function_2021	1	1	1	1	1	1	1	1	2	3	7	11	14	17	23	29	39	42	48	47	61
		KEGG_2019_Mouse	1	1	1	1	1	1	1	1	2	3	7	8	14	15	21	27	25	27	29	29	33
		Reactome_2016	1	1	1	1	1	1	1	1	2	3	6	11	18	20	29	38	33	46	43	45	53
	**Pando**	GO_Biological_Process_2021	1	1	2	1	2	2	2	2	2	2	2	12	9	15	15	12	13	19	15	11	10
		GO_Cellular_Component_2021	1	2	1	1	1	2	2	2	2	2	2	5	5	9	6	7	8	10	7	7	3
		GO_Molecular_Function_2021	1	2	3	2	2	2	2	2	2	2	2	14	16	14	14	16	15	23	17	17	17
		KEGG_2019_Mouse	1	2	2	1	2	2	2	2	2	2	2	9	8	9	9	8	8	9	7	7	4
		Reactome_2016	1	2	2	1	2	2	2	2	2	2	2	11	9	13	6	11	10	12	9	9	6
	**GENIE3**	GO_Biological_Process_2021	1	1	1	1	1	2	2	3	3	3	4	5	5	5	7	8	8	10	8	14	12
		GO_Cellular_Component_2021	1	1	1	1	1	2	2	3	3	3	4	5	5	5	5	7	10	7	10	12	10
		GO_Molecular_Function_2021	1	1	1	1	1	2	2	3	3	3	4	5	5	6	6	8	7	7	9	13	13
		KEGG_2019_Mouse	1	1	1	1	1	2	2	3	3	3	4	5	5	5	7	7	7	8	9	10	7
		Reactome_2016	1	1	1	1	1	2	2	3	3	3	4	5	5	6	5	7	8	8	8	13	10
	**HuMMuS + TF**	GO_Biological_Process_2021	1	1	1	1	1	1	1	1	3	6	10	11	13	18	23	27	30	37	33	35	40
		GO_Cellular_Component_2021	1	1	1	1	1	1	1	1	3	5	8	9	7	8	12	12	17	18	15	18	20
		GO_Molecular_Function_2021	1	1	1	1	1	1	1	1	3	6	10	12	13	17	23	23	29	35	32	32	36
		KEGG_2019_Mouse	1	1	1	1	1	1	1	1	2	4	8	9	11	13	16	18	21	21	23	25	25
		Reactome_2016	1	1	1	1	1	1	1	1	3	6	10	12	12	15	20	23	24	24	25	27	30
	**HuMMuS**	GO_Biological_Process_2021	1	1	1	1	1	1	1	1	2	3	7	14	15	19	24	29	29	32	37	41	46
		GO_Cellular_Component_2021	1	1	1	1	1	1	1	1	2	3	5	9	8	11	14	11	14	15	16	17	25
		GO_Molecular_Function_2021	1	1	1	1	1	1	1	1	2	3	8	14	16	17	24	27	32	31	34	40	35
		KEGG_2021_Human	1	1	1	1	1	1	1	1	2	3	5	10	11	14	16	18	22	22	24	27	24
		Reactome_2016	1	1	1	1	1	1	1	1	2	3	8	12	14	19	24	24	26	31	31	34	33
	**SCENIC+**	GO_Biological_Process_2021	1	1	1	1	1	1	2	2	3	2	5	7	5	7	9	11	13	17	21	24	28
		GO_Cellular_Component_2021	1	1	1	1	1	1	2	2	3	3	5	5	5	5	8	13	15	15	20	22	17
		GO_Molecular_Function_2021	1	1	1	1	1	1	2	2	3	1	5	6	6	5	10	13	19	20	19	22	36
		KEGG_2021_Human	1	1	1	1	1	1	2	2	3	2	5	5	7	9	8	7	10	14	14	20	13
		Reactome_2016	1	1	1	1	1	1	2	2	2	2	5	5	6	5	10	10	10	18	12	19	22

Supplementary Table 6. Marker TFs/regulons identified by HuMMuS on the cortical mouse 3-omics dataset. This table contains the regions and the Wilcoxon test's statistics associated to the identified marker TFs, and the literature supporting their levels of evidence. Different level of literature-based evidence are indicated by colors : green (marker of the specific subpopulation supported by literature), blue (marker of the cortex or similar area supported by literature), yellow (other brain regions / neuron markers supported by literature), orange (expressed in the brain/cortex according to gene expression databases), gray (no evidence)

gene name	area	Wilcoxon test score / p-values adjusted	associated publication / source
Tbx1 Tbx10	Layer 2/3	104.65	loss of Tbx1 disrupts corticogenesis in mice by promoting premature neuronal differentiation
		0	https://pubmed.ncbi.nlm.nih.gov/27005988/
Rfx3	Layer 2/3	102.37	*We [...] identified [...] TFs with more restricted patterns in specific subclasses, such as Rfx3 [...] (in L2/3 IT)*
		0	A multimodal cell census and atlas of the mammalian primary motor cortex
Vdr (vitamin D)	Layer 2/3	102.2	Expressed in cortical neurons and involved in neurodegeneration
		0	https://pubmed.ncbi.nlm.nih.gov/21408608/
Neurog1	Layer 2/3	98.26	layer II/III neurons of the piriform cortex.
		0	https://pubmed.ncbi.nlm.nih.gov/24403153/
Zfp711	Layer 2/3	96.2	not studied a lot, involved in brain development
		0	https://pubmed.ncbi.nlm.nih.gov/20346720/
Pou4f1 Pou4f2	Layer5a	81.53	Expressed in [...] the dorsal column of the mesencephalic and pontine central gray, and the lateral interpeduncular nucleus of the brain
		0	https://pubmed.ncbi.nlm.nih.gov/7904822/
Esx1	Layer5a	76.13	expressed highly in midbrain mantle layer (FDR: 4E-4)
		0	https://bgee.org/gene/ENSMUSG00000023443?expression=&data_type=IN_SITU
Sebox	Layer5a	71.97	expressed in cerebral cortex
		0	https://www.ncbi.nlm.nih.gov/pmc/articles/PMC16794/
Setbp1	Layer5a	70.6	expressed in ventricular zone
		0	https://molecularautism.biomedcentral.com/articles/10.1186/s13229-023-00540-x
Pou4f3	Layer5a	70.2	Expressed in brain and DRG
		0	https://pubmed.ncbi.nlm.nih.gov/22326227/
Pgr	Layer5	79.42	expressed in substantia niagra
		0	https://bgee.org/gene/ENSMUSG00000031870
Nr3C1	Layer5, Layer6	76.48, 75.96	expressed in median eminence of neurohypophysis
		0, 0	https://bgee.org/gene/ENSMUSG00000024431
Mbd2	Layer5, MGE, Layer5b	67.94, 60.58, 54.90	highly expressed in brain
		0, 0, 0	https://pubmed.ncbi.nlm.nih.gov/9774669/
Wt1	Layer5, CGE, MGE	63.45, 71.84, 60.58	neurons of DRG and sertolis cells
		0, 0, 0	https://pubmed.ncbi.nlm.nih.gov/16467207/
Pbx2 /Pbx4	Layer5	54.88	regulates patterning f the cerebral cortex in progenitors and post mitotic neurons
		0	https://pubmed.ncbi.nlm.nih.gov/26671461/
Olig3	Claustrum	29.18	**Olig3 coordinates the specification of dorsal neurons in the spinal cord**
		3.54E-187	https://www.ncbi.nlm.nih.gov/pmc/articles/PMC1065726//
Foxn2	Claustrum	27.27	
		9.80E-164	
Naif1	Claustrum	27.09	
		1.23E-161	
Dmrtc2	Claustrum	26.7	*DMRT2, DMRTA1/DMRT4, DMRT3 and DMRTA2/DMRT5 are expressed mainly in cortical regions*
		4.58E-157	https://www.frontiersin.org/articles/10.3389/fnana.2022.937596/full
Cdc5l	Claustrum	26.61	
		5.31E-156	
**Maz**	**CGE**	73.36	(1) Expressed in Purkinje cells in the brain (at protein level). / (2) driving neurogenesis
		0	(1) https://pubmed.ncbi.nlm.nih.gov/26089202/ (2) https://pubmed.ncbi.nlm.nih.gov/22944911/
Sp2	CGE	72.6	CHECK AGAIN
		0	
Smad1/Smad5	CGE, MGE	71.92, 60.57	ventricular zone, FDR: 10E-10
		0, 0	https://www.uniprot.org/uniprotkb/P97454/entry#expression
Zbtb1	CGE	70.19	
		0	
Zfp219	MGE	60.28	
		0	
Klf15	MGE	60.05	
		0	
Mlxipl	Layer5b	48.23	Expressed in the ventricular and intermediate zones of the developing spinal cord of 12.5 dpc embryos. In later embryos expressed in a variety of tissues.
		0	https://www.uniprot.org/uniprotkb/Q99MZ3/entry#expression
Hoxa1	Layer5b	48.19	Motor neuron axon guidance in development
		0	https://pubmed.ncbi.nlm.nih.gov/9367425/
Arntl	Layer5b	46.21	constitutively expressed in hypothalamus nucleus suparchiasmatic
		0	https://pubmed.ncbi.nlm.nih.gov/11207387/
Mitf	Layer5b	46	Microphthalmia-associated transcription factor ensures the elongation of axons and dendrites in the mouse frontal cortex.
		0	https://pubmed.ncbi.nlm.nih.gov/27859996/
Mnt	Layer6	80.83	Motor neuron expression
		0	https://bgee.org/gene/ENSMUSG00000000282
Zfp282	Layer6	78.68	
		0	
Lef1	Layer6	75.96	deep layers of the cortex, important for normal development
		0	https://www.ncbi.nlm.nih.gov/pmc/articles/PMC3825142/
Tcf7l2	Layer6	74.89	deep layers of the cortex, important for normal development
		0	https://www.ncbi.nlm.nih.gov/pmc/articles/PMC3825142/

Supplementary Table 7. Resources used to run HuMMuS workflow on different datasets.

		Mouse Cortex Dataset	Chen Dataset
	Size scRNA seq data	25299 genes; 55803 cells	8595 genes; 385 cells
	Size scATAC seq data	155093 peaks; 2317 cells	36954 peaks; 385 cells
Multilayer Creation	Real time	2 h 30 min	41 min 9 sec
	Memory used	50 Gb	26 Gb
	Number of CPUs	90	90
	CPU time	23 h 5 min	3 h 40 min
TF - target genes/regions	Real time per TF (RWR process)	1 min 35 sec	7.51 sec
	Real time all TFs	28 min 25 sec	3 min 58 sec
	Memory used	270 Gb	32 Gb
	Number of CPUs	70	70
	CPU time	24 h 40 min	1 h 47 min
TF - target genes/regions	Real time per TF (RWR process)	1 min 40 sec	8.27 sec
	Real time all TFs	9 h 20 min	10 min 02 sec
	Memory used	270 Gb	32 Gb
	Number of CPUs	70	70
	CPU time	548 h 10 min	10 h 44 min
